# Design of a New Chiral Deep Eutectic Solvent Based on 3-Amino-1,2-propanediol and Its Application in Organolithium Chemistry

**DOI:** 10.3390/molecules27238566

**Published:** 2022-12-05

**Authors:** Achille Antenucci, Matteo Bonomo, Simone Ghinato, Marco Blangetti, Stefano Dughera

**Affiliations:** 1 Department of Chemistry, Università degli Studi di Torino, Via Pietro Giuria 7, 10125 Torino, Italy; 2 Centro Ricerche per la Chimica Fine s.r.l. for Silvateam s.p.a., Via Torre 7, 12080 San Michele Mondovì, Italy; 3 NIS Interdepartmental Centre and INSTM Reference Centre, Università degli Studi di Torino, Via Gioacchino Quarello 15/a, 10125 Torino, Italy

**Keywords:** deep eutectic solvent, organolithium reagents, 3-amino-1,2-propanediol

## Abstract

A chiral glycerol derivative, namely 3-amino-1,2-propanediol, was employed for as the hydrogen bond donor (HBD) in the design of a new deep eutectic solvent (DES) with choline chloride acting as the hydrogen bond acceptor (HBA). The novel mixture was characterized and unambiguously classified as a DES. Furthermore, its synthetic usefulness was demonstrated in the room-temperature *n*-butyllithium-addition under air to carbonyl compounds and benzyl chloride. In some cases, pure products (100% conversion) were obtained by a simple extractive work-up in up to 72% isolated yield, thus suggesting the potential practical usefulness of this procedure as a green alternative to the classical Schenk procedure in volatile organic solvents for the synthesis of tertiary alcohols. The chirality of the HBD, bearing an interesting basic primary amino group, is an intriguing feature currently under investigation for further exploitation.

## 1. Introduction

Starting from the end of the 20th century, the growing attention towards environmental sustainability and the health and safety of laboratory operators has started to inspire different branches of chemistry, including synthetic organic chemistry. In particular, one among the 12 principles of green chemistry highlights the necessity of developing greener alternatives to classic volatile organic compounds (VOCs), still nowadays routinely employed both as reaction media and nonaqueous solvents for several work-up procedures [1,2,3]. Since their first appearance in 2003 [4], deep eutectic solvents (DESs) have emerged as a suitable alternative to VOCs [5,6,7,8] for diverse practical applications including, among others, drug delivery [9,10,11], electrochemistry [12,13,14,15], material design [16], extractions [17] and, of course, organic reactions [18], alone or together with other sustainable reaction-media [19,20]. In the context of organic synthesis, desirable features of DESs include (i) low vapor-pressure [21], (ii) chemical stability, (iii) ease of design and preparation in the absence of any VOC, and (iv) handy separation of the reaction crude by simple precipitation or aqueous work-up [22]. Last but not least, the handiness of organic compounds and even their reactivity in DESs have often been found to be surprising, offering food for thought from a mechanistic point of view [23,24,25,26].

**Figure 1:**
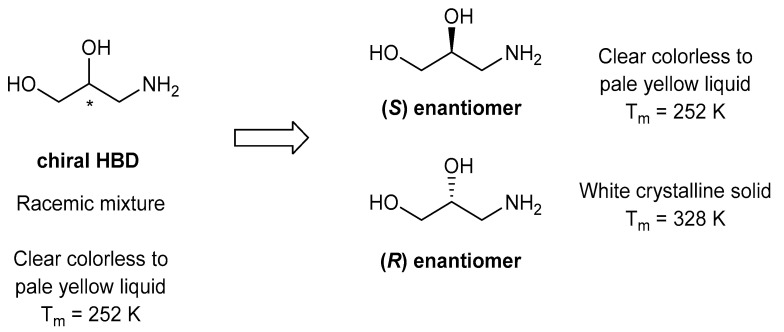
3-Amino-1,2-propanediol and its chemico-physical properties as an HBD for the novel DES.

Differently from ionic liquids, in which electrostatic interactions play a major role, molecular interactions involved in the formation of DESs mainly entail the establishment of a robust hydrogen-bond network, besides electrostatic and Van der Waals interactions. Consequently, DESs are commonly prepared by mixing and/or heating a proper hydrogen bond donor (HBD) and a suitable hydrogen bond acceptor (HBA) [6]. However, the unambiguous classification of such kind of mixture as a true DES is anything but obvious [26], unless two room-temperature solid components lead to a room-temperature liquid mixture. Following on from our previous studies, we managed to design, prepare and characterize a new DES by introducing an unexplored HBD, namely 3-amino-1,2-propanediol (Figure 1), herein referred to as aminoglycerol (AGly). With respect to glycerol, an HBD commonly found as a DES constituent, the replacement of a primary hydroxyl group with an amino group could likely introduce both basic properties and chirality in the eutectic mixture. With this purpose, it must be stressed that a systematic study of asymmetric induction by chiral DESs as reaction media, and therefore their exploitation as a general strategy in asymmetric synthesis, are still missing. These two possibly desirable features open new and intriguing scenarios in the reactivity of organic substrates in DESs, which are under investigation in ongoing studies.

Herein, the preparation and physico-chemical characterization of the eutectic mixture, AGly/choline chloride 3:1, is presented, along with its synthetic usefulness as a reaction medium. Recently, our research group has contributed to growing the chemical portfolio of air- and moisture-compatible highly polar *s*-block organometallic reactions, refusing the historical paradigm that organolithium chemistry should always be performed under strict, tedious and energy-consuming Schlenk-type reaction conditions [23]. In particular, ChCl-based DESs have shown promise as environmentally sustainable reaction media to promote chemo- and regioselective metalations and nucleophilic-acyl-substitution reactions, using highly reactive alkyl-lithiums, working at room temperature and under air, with an unprecedented high level of chemocontrol, compared with the classical methodology [24,25,26]. Thus, our new synthesized DES AGly/choline chloride 3:1 is used as a reaction medium to promote the environmental-beneficial addition of *n*-BuLi (as a model organolithium compound) to several carbonyl compounds including benzyl chloride, whose unusual reactivity in the nucleophilic substitution reaction with organolithium reagents had never been successfully performed in protic media (among which are DESs), until now.

## 2. Results and Discussion

Despite a multitude of DESs having been designed and employed in a plethora of different applications, general criteria for an unambiguous classification of DESs, and thus for their design by default, are still lacking. This is also due to the fact that a thorough knowledge of DES structure (i.e., the chemical and physical reason behind the formation of a eutectic mixture), is still far from being reached [27]. Consequently, many of the mixtures employed as reaction media in organic synthesis cannot be strictly defined as DESs, but rather as low-melting-temperature mixtures (LMTMs) or, more generally, unconventional solvents [6]. In order to provide a solid starting point for the design of a novel DES, we chose the system glycerol/choline chloride (Gly/ChCl) as a model system, and then slightly modified the structure of the HBD. Indeed, it has been previously demonstrated that both a 2:1 and 3:1 ratio of glycerol/choline chloride afford the establishment of a complete coordination shell of hydroxyl groups around the chloride anion, either belonging to the sole HBD (3:1 ratio) or belonging both to the HBD and the choline moiety (2:1 ratio), with the former showing a lower melting-temperature and thus claiming the role of a pure eutectic mixture [28]. Glycerol is a prochiral molecule, and the replacement of a primary hydroxyl with an amino group introduces chirality as an intriguing and exploitable feature for synthetic chemistry, without completely removing a hydrogen-bond-donor site, as it would instead result from the introduction of an aprotic substituent. Nevertheless, in as far as the amino group might potentially contribute to the establishment of a (different) hydrogen-bond network, its behavior with respect to a hydroxyl group could not be predicted *a priori*. Therefore, four mixtures of AGly/ChCl are prepared by mixing (353 K, 30 to 60 min) the racemic form of the HBD with the HBA in 1:1, 2:1, 3:1 and 4:1 ratios.

**Figure 2:**
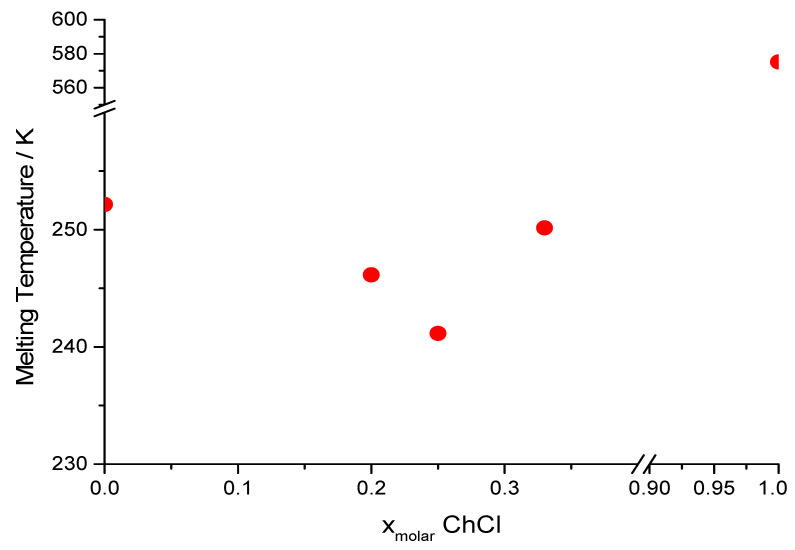
Experimental phase-diagram of AGly and ChCl mixtures built up by using the T_m_ values of the precursor available in the literature (confirmed in our labs) and the experimental T_m_ values of the mixtures as an average of the values obtained from the different approaches reported in the experimental section. The error bar is lower than 2 K for all the mixtures.

As a matter of fact, the behavior of the mixtures is quite different: indeed, a 1:1 ratio leads to a solid system, even if the temperature is set well above 353 K. Conversely, when the amount of AGly is increased, the mixtures become homogenous liquids after gentle heating. Unfortunately, once cooled down to RT, the 2:1 mixture forms a considerable amount of precipitate (likely ChCl crystals) within a few hours, discouraging us to further investigate it as a solvent (*vide infra*). Both the 3:1 and 4:1 ratios allow the formation of a homogeneous liquid mixture at RT. Yet, by simple inspection of the obtained liquid mixture, the identification of the real eutectic composition is far from straightforward, especially considering that one of the precursors (i.e., AGly, racemic) is liquid at RT (T_m_ = 252 K). With this purpose, the obtainment of a liquid mixture starting from *(R)*-AGly (solid) can, however, be considered rough evidence of the obtainment of an eutectic mixture. A valuable aid is given by the discussion of T_m_ values of the obtained mixture. The latter is usually extracted from DSC (differential scanning calorimetry), yet it is well known [29] that glycerol-like systems behave like glass, showing a nearly zero enthalpy melting/crystallization. In our case, we are able to measure a glass transition (in the heating cycle) in both the 3:1 and 4:1 systems (Figure S1 in the Supplementary Material), and we firstly ascribe it to the T_m_ values of the mixtures (i.e., 241 K and 246 K, respectively). To further confirm these data, we resolved to manually measure the T_m_ (Table 1), as described in our previous paper [30]; the value for racemic AGly was also measured and found to be in excellent agreement with the literature data (±2 K). These data allow us to draft an experimental phase-diagram of the AGly and ChCl mixtures (Figure 2), proving the 3:1 mixture as the DES.

**Figure 3:**
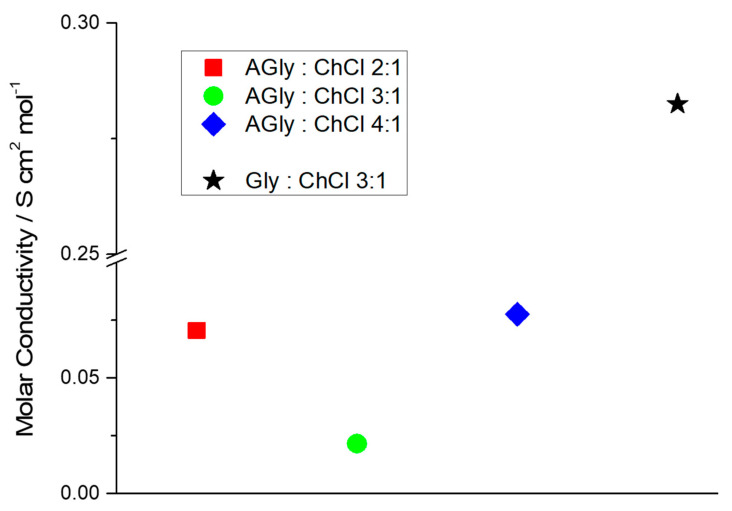
Molar conductivity of the mixtures AGly:ChCl 2:1 (red square), 3:1 (green circle) and 4:1 (blue diamond) at RT. The value of a DES based on Glycerol and ChCl (3:1) is reported as a comparison (black star).

Screening the literature [6], the formation of ChCl-based DESs is conventionally ascribed to the stabilization of the Cl^−^ anion by the instauration of a hydrogen-bond network with the HBD moieties, e.g., hydroxyl, aminic). Such stabilization will lead to a modification of some critical physico-chemical properties of the mixtures such as viscosity (η) and molar conductivity (Λ). Yet, these modifications are difficult to predict, being strongly dependent on the nature of both the HBD and the HBA [31]. Thus, to further prove the formation of a non-conventional mixture, we measured both η and Λ of all the systems, except AGly:ChCl 1:1 (being solid). Experimental results highlight the fact that whereas η follows a monotonic trend, i.e., it decreases with the increase of the HBD amount, Λ shows a peculiar trend, characterized by a minimum for AGly:ChCl 3:1 (22 mS cm^−1^ M^−1^, Table 1). Even more interestingly, both AGly:ChCl 2:1 and AGly:ChCl 4:1 have an almost identical value of Λ (Figure 3), proving that the charge carriers (i.e., Cl^−^ anions) experiment a similar chemical environment (i.e., hydrogen-bond network). Thus, the lower molar-conductivity of AGly:ChCl 3:1 could be easily rationalized with a more extended HB-network, typical of a DES, which limits the mobility of the chloride. This could be considered as valuable information toward the application of this DES as a solvent in Li-promoted organic reactions: indeed, a hydroxyl moiety strongly involved in a hydrogen bond will be quite refractive toward the (unwanted) reaction with the catalyst, leading to a safer and more controllable reaction.

The higher molar-conductivity and the lower viscosity (not shown) of the reference DES based on glycerol (i.e., Gly:ChCl 3:1) is critical evidence for postulating that the amino moieties of aminoglycerol play only a limited role in the stabilization of the Cl^−^ (as expected from their low acidity).

Strong evidence of the formation of a real eutectic is given by the obtainment of a room-temperature-liquid DES also by mixing, in a 1:3 ratio, ChCl (solid) with enantiopure (*R*)-AGly that, being different from its racemic form and the (*S*)-enantiomer (colorless viscous liquids), is a crystalline white solid with a melting point of 328 K [32].

In order to demonstrate the synthetic usefulness of this novel DES, we decided to test its ability to promote the chemoselective addition of *n*-BuLi to highly reactive carbonyl compounds. This reaction was very well known as the first use of protic DES as reaction media in the chemistry of highly reactive *s*-block organometallic species [23]. Thus, in a preliminary experiment, 2′-methoxyacetophenone **1a** (1 mmol) was suspended, both in the Gly/ChCl 2:1 (1 g) eutectic mixture (Table 2, entry 1) and in AGly/ChCl 3:1 (Table 2, entry 2); it was then reacted at room temperature under air, with a commercial 2.5 M hexanes solution of *n*-BuLi (2 equiv.) under continuous stirring. To our delight, the yields of the desired tertiary alcohol **2a** obtained after work-up using these two different DESs, were comparable (Table 2, entries 1–2). Moreover, an important yield-increase could be observed when increasing the amount of organolithium reagent from 2.0 to 3.0 equivalents (Table 2, entry 3). It must be stressed that the possible deprotonation of the NH_2_ group by *n*-BuLi did not occur, as no nucleophilic-addition product was ever detected. Interestingly, in the reaction carried out in pure AGly (Table 2; entry 4) the conversion was not total, as 33% of unreacted **1a** remained, in contrast with what is observed if the reactions are performed in DES (Table 2; entry 3).

**Scheme 1:**
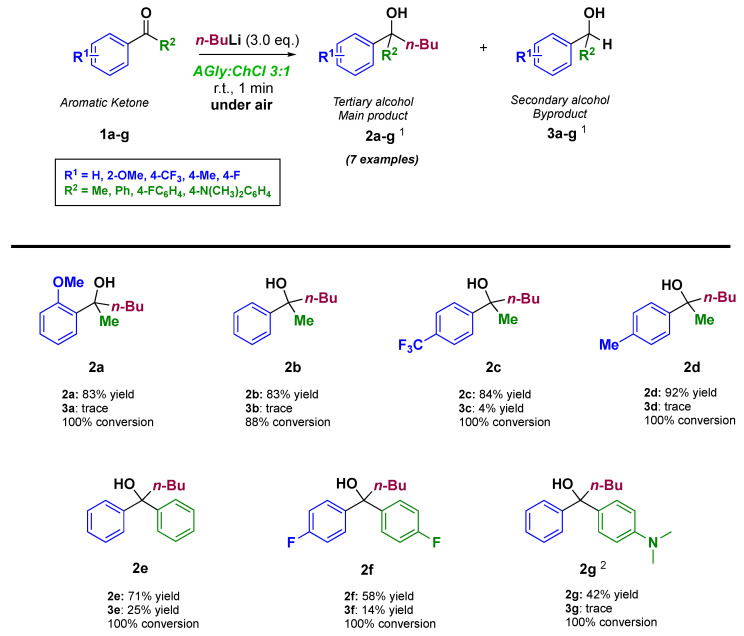
Substrate scope of the reactions of aromatic ketones with *n*-BuLi. ^1^ Reaction conditions: **1a**–**g** (1 mmol), *n*-BuLi (2.5 M in hexanes, 3 mmol), DES (1.0 g), under air at room temperature. The yields of products **2** and **3** reported are determined by ^1^H NMR, using CH_3_NO_2_ as the internal standard. ^2^ 2.0 equiv. of *n*-BuLi.

Encouraged by this finding, we assessed the reactivity of some different classes of carbonyl compounds in such a medium (Scheme 1). An exemplary series of aromatic ketones, namely acetophenones **1a**–**d** and benzophenones **1e**–**1g,** bearing both EDG **1a**, **1g**, EWG **1c**, **1f** and the neutral functional-group **1b**, **1d**, **1e,** were reacted with three equivalents of *n*-BuLi in AGly:ChCl 3:1, giving the desired tertiary alcohol **2** in good to quantitative yields (up to 92%). Notably, in all cases the reaction maintained an elevated level of selectivity and no traces of lithiation byproducts were observed, corroborating our previous observation [24] about the attitude of the eutectic mixture to exalt, probably via ammonium salt (ChCl) coordination [23], the nucleophilic behavior of *n*-BuLi under these bench-type reaction conditions. Furthermore, in all cases, only a small amount of a secondary alcohol **3** was observed as a byproduct arising from an *n*-BuLi-promoted carbonyl reduction event. Finally, in the case of product **2g**, no yield improvement was observed by increasing the organolithium amount from 2.0 to 3.0 equivalents and, due to the low conversion, only traces of the undesired byproduct **3g** could be observed in the ^1^H NMR spectrum.

**Scheme 2:**
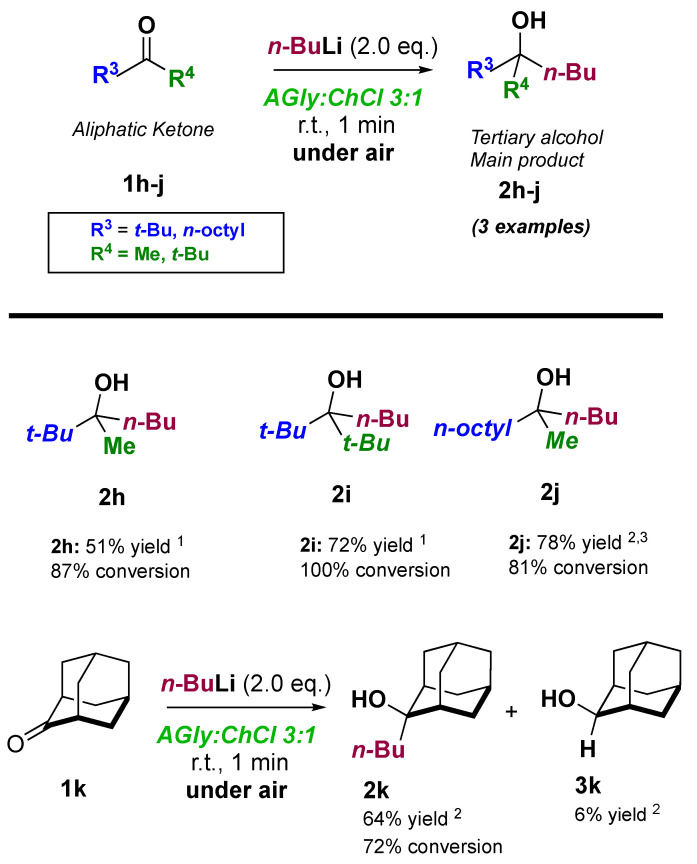
Substrate scope of the reactions of aliphatic ketones with *n*-BuLi. ^1^ Isolated yields. ^2^ NMR yields confirmed by GC. ^3^ 3.0 eq. of *n*-BuLi.

At this stage, the protocol was extended to a series of aliphatic ketones **1h**–**k** (Scheme 2) that are notoriously challenging substrates, due to their sensitivity to the enolization. In this case, conversion values were generally higher (up to 100%) with respect to aromatic ketones, but at the same time the yields were systematically lower, a trend exclusively due to the volatility of the involved species (see Supplementary Materials). Notably, the elevated steric hindrance of all the employed *n*-BuLi aliphatic substrates **1h, 1i, 1k** did not limit the successful outcome of the nucleophilic addition of the organolithium. A lower amount of *n*-BuLi (2.0 equiv.) was sufficient to reach the optimum, and no yield increase was observed upon increasing the excess of the organolithium reagent for tertiary alcohols **2h** and **2i**. Notably, product **2i** could be isolated in pure form by means of a simple extractive work-up in 72% yield. On the other hand, the conversion of open-chain 2-decanone **1j** and sterically hindered 2-adamantanone **1k** benefited from a higher amount (3.0 molar equivalents) of *n*-BuLi (Scheme 2). As mentioned before, the reaction showed an appreciable level of selectivity, and only a small amount of secondary alcohol was observed as a byproduct when 2-adamantanone **1k** was used as substrate, while no enolization byproducts were observed using enolisable ketones **1i** and **1j,** confirming the promising attitude of *n*-BuLi as a nucleophilic species under this thermodynamic reaction condition [22].

**Scheme 3:**
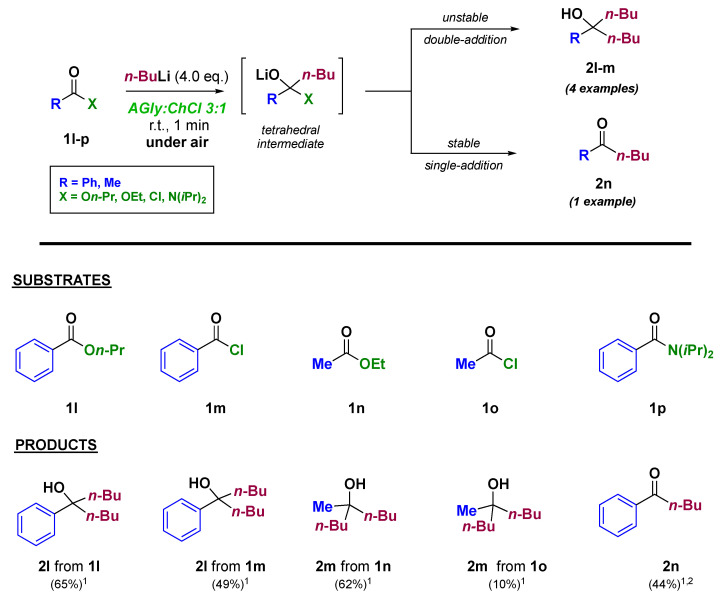
Substrate scope of the reaction of carboxylic-acid derivatives (esters, acyl chlorides, amides) with *n*-BuLi. ^1^ Reaction conditions: **1l–o** (1 mmol), *n*-BuLi (2.5 M in hexanes, 4 mmol), DES (1.0 g), under air, room temperature. The yields reported are for products isolated after flash column chromatography on silica gel. Conversions were 100%. ^2^ Reaction conditions: **1p** (1 mmol), *n*-BuLi (2.5 M in hexanes, 2 mmol), DES (5.0 g), CPME (1 mL), under air, 0 °C. 11% of tertiary alcohol was detected. Conversion was 100%.

To our delight, in addition to these results, we also found out that the reaction of esters and acyl chlorides with 4.0 molar equivalents of *n*-BuLi in DES AGly:ChCl 3:1 afforded the corresponding tertiary alcohols (resulting from a double nucleophilic attack of the organolithium reagent upon the collapse of the in situ generated tetrahedral intermediate) in pure form. It must be stressed that, to the best of our knowledge, the reactivity of acyl chlorides had not been previously assessed in DESs, while that of esters has only recently been explored by Capriati and co-workers [33]. Pure 5-phenyl-5-nonanol **2l** could be isolated in 65% yield from propyl benzoate **1l** and in 49% yield from benzoyl chloride **1m**, while the *n*-BuLi double addition to ethyl acetate **1n** and acetyl chloride **1o** afforded pure 5-methyl-5-nonanol **2m** in 62% and 10% yield, respectively. Finally, the unconventional reactivity of nucleophilic addition-resistant sterically hindered carboxylic acid amides recently shown by Ghinato et al. towards organolithium reagents [26] was s confirmed also in AGly:ChCl 3:1. Indeed, the treatment of *N,N*-diisopropylbenzamide **1p** with 2.0 equivalents of *n*-BuLi afforded tertiary alcohol **2l** only in 11% yield, while the major product was ketone **2n**, whose yield was 44% (Scheme 3), due to the higher stability of the amide-derived tetrahedral intermediate, compared with the same obtained from esters and acyl chlorides. This result confirms the excellence of bench-stable and easy-to-handle carboxylic acid amides as an acylation agent of organolithiums reagents, to access functionalized ketones in a chemoselective manner.

The transfer of chirality from the reaction medium to the products is still a major challenge in the case of DESs; good to excellent results were afforded only in isolated cases [34,35], and lacking a general mechanism which could make this a general strategy in asymmetric synthesis. Therefore, with a novel optically active DES in our hands, we managed to perform the model reaction of substrate **1a** in optically active DES *(R*)-AGly:ChCl 3:1 (reaction conditions described in Scheme 1). Unfortunately, no enantiomeric excess was observed by HPLC in the chiral stationary phase. We speculate that the reaction of the organolithium reagents is too fast to allow the amino group of the HBD to coordinate the lithiated species, providing the basis for chirality transfer.

Encouraged by these results, we decided to approach aliphatic nucleophilic substitution with organolithium reagents in this newly designed DES, at room temperature. It is well known that nucleophilic substitutions occur in aprotic solvents (either non-polar or polar), and that highly basic organometallic reagents do not act as good nucleophiles towards alkyl halides. However, some examples of unconventional nucleophilic reactivity of organometallic compounds towards benzyl halides and other aliphatic halides have been reported in the literature, including the reactivity of *n*-BuLi [36] and 9-phenanthrylmagnesium bromide [37] towards benzyl chloride, the reactivity of Grignard reagents towards sulfur mustards [38], the reactivity of *n*-BuLi towards 1-bromo-2-phenethylbenzene [39], and the reactivity of *t*-BuLi towards benzyl chloride [40,41]. These transformations, classifiable as examples of the Wurtz reaction [42,43], were performed in apolar (hexanes, benzene, toluene) or low polar (Et_2_O, THF) aprotic solvents, at low temperatures (−100 to 0 °C).

We decide to explore the reactivity of benzyl chloride **4** with *n*-BuLi in different DESs (Table 2), including the eutectic mixture Gly:KF 6:1 (Table 2, entry 4), recently designed and exploited by some of us [44,45,46].

Excluding the water-based mixture H_2_O:ChCl 2:1 (Table 3, entry 7), in which no conversion was observed, due to the rapid *n*-BuLi protonation, the reaction occurred in all of the tested reaction media. The best results (85% yield) were observed when AGly:ChCl 3:1 was used as the reaction medium (Table 3, entry 4). Despite the possible formation of in situ AGly lithium amide and relative nucleophilic displacement on **4**, only traces of the corresponding reaction byproduct 3-(benzylamino)propane-1,2-diol **7** could be observed. It must be stressed that the reaction carried out in AGly/ChCl had a noteworthy yield increase with respect to the corresponding Gly-containing eutectic mixture (Table 3, entries 1-2,6) and to other HBD-based-DESs, such as urea (Table 3, entry 3), lactic acid (Table 3, entry 5) and ethylene glycol (Table 3, entry 8). For all these reactions, the only byproduct was 1,2-diphenylethane **6**, deriving from the *n*-BuLi-promoted homocoupling of two equivalent to **4**. Therefore, we can state that the best results refer to the engineered DESs, whose design and rigorous chemico-physical characterization was performed by us, corroborating the importance of DES design.

Furthermore, it must be stressed that this is also, to the best of our knowledge, the first example of aliphatic nucleophilic substitution promoted by an organometallic reagent in protic reaction-media under sustainable reaction conditions DES [47,48,49], which will be more deeply investigated as the subject of a forthcoming paper.

## 3. Materials and Methods

**General.** All reagents and solvents were purchased from commercial sources (Sigma Aldrich, Alfa Aesar, TCI, Fluorochem) at the highest-available purity grade, and used without further purification, upon checking their purity by NMR, TLC or GC-MS. *n*-Butyllithium was purchased from Sigma Aldrich in hexanes solution (rated concentration 2.5 M) and titrated with diphenylacetic acid in anhydrous tetrahydrofuran. Preliminary trials of addition to carbonyl compounds were performed in duplicate with different batches of this organolithium reagent. Thin layer chromatography was performed on Merck silica gel TLC plates GF 254 on aluminum support. All the reactions were carried out in open air glassware and monitored by GC-FID, GC-MS and NMR. Mass spectra were recorded on an HP 5989B mass selective detector connected to an HP 5890 GC with a methyl silicone capillary column. GC-FID analyses were performed on a Perkin Elmer AutoSystem XL GC with a methylsilicone capillary column. ^1^H NMR and ^13^C NMR spectra were recorded in CDCl_3_ (solvent residual peak at δ = 7.26 ppm for ^1^H NMR and 77.16 ppm ^13^C NMR) or in CD_3_OD (solvent residual peak at δ = 3.31 ppm for ^1^H NMR and 49.00 ppm for ^13^C NMR) on a Jeol ECZR spectrometer at 600 and 150 MHz, respectively, and using diazomethane as an internal standard for the calculation of the NMR yields. Chemical shifts are expressed in ppm and referred to solvent residual peaks. HPLC analyses on the chiral stationary phase were performed using a Waters 1525 HPLC pump provided with a Waters 2998 UV-VIS spectrofotometer with photodiode array (PDA) detector on a Daicel CHIRALPAK^®^ IC column (250 × 4.6 mm, 5 μm), using mixtures of hexanes and 2-propanol.

Viscosity measurements were performed on an Anton Paar DV-1 P viscometer at room temperature and atmospheric pressure.

Conductivity measurements were carried out using a conductivity electrode (5072 from Crison, K = 10 cm^−1^) with platinum plates constituting both the working and the counter electrodes. The electrode was coupled to an Autolab potentiostat/galvanostat Model PGSTAT12^®^ from Metrohm, remotely controlled by the computer using ECLab software (v11.20). An electrochemical impedance (EIS) scan was employed to measure the A.C. resistance of each solution, from which the relative conductivity was calculated. In the EIS experiments the amplitude of the potential perturbation was set to 50 mV. Impedance spectra were recorded within the frequency ranges 10^3^−10^7^ Hz.

Differential scanning calorimetry (DSC) measurements were conducted through a DSC Q200 (from TA Instrument) equipped with a cooling flange. A drop (ca. 20 mg) of each mixture was sealed in an aluminum pan and cooled from RT to 190 K. After being at that temperature for 20 min, they were heated up to 303 K with a heating ramp of 2 K/min and then cooled back at 190 K, with the same ramp. A homemade set-up (i.e., a vial containing the mixture capped with a thermometer) was also employed to confirm the DSC data. The set-up was stored in a fridge at 188 K for 24 h. At this stage, the mixture was solidified around the thermometer tip. Once taken out of the fridge, the system was kept at 298 ± 1 K and the melting temperature was estimated as the one at which the thermometer could be extracted from the vial. Each measurement was repeated three times, with a standard deviation lower than 1.5 K. When available, the comparison between the Tm values calculated using these three methods led to a difference lower than 2 K.

All the mixtures employed in the present study were prepared by mixing the quaternary ammonium salt choline chloride (ChCl) with the proper amount of the corresponding hydrogen bond donor (glycerol (Gly) or aminoglycerol (AGly). Deep eutectic solvents based on glycerol and choline chloride were prepared according to the literature and used without further purification.

**Synthesis of DES AGly:ChCl 3:1.** To a round-bottom flask, 37.88 mmol (5.29 g) of choline chloride (ChCl, white solid) and 113.60 mmol (10.35 g) of aminoglycerol (AGly), colorless viscous liquid in the racemic form or in the enantiopure form (*S*), white crystalline solid in the enantiopure form (*R*)) were added. The resultant mixture was heated up to 80 °C for 30 min, to yield a clear solution. The obtained DES AGly:ChCl 3:1 was used without further purifications.

**General procedure for the addition of *n*-butyllithium to carbonyl compounds in DESs.** Reactions were performed at room temperature and under air, unless otherwise specified. In a glass tube, the appropriate carbonyl compound (1 mmol) was dissolved in the corresponding deep eutectic solvent (DES, 1 g) under air, followed by stirring for 5 min in order to obtain a homogeneous mixture. In the case of the solid substrates, the mixture was heated to 50 °C for 5 min under stirring, to obtain a homogenous mixture. The selected amount of *n*-butyllithium (1 to 4 mmol, 2.5 M in hexanes) was then added at room temperature and under vigorous stirring, and the reaction mixture was stirred for 1 min. The addition of water (20 mL) to the reaction crude, followed by extraction with ethyl acetate (3 × 10 mL) and drying of the organic phase over Na_2_SO_4_ and then evaporation under vacuum, allowed the isolation of the crude product. The identity of the tertiary alcohols **2a** [50], **2b** [51], **2d** [52] **2e** [53], **2g** [54], **2h** [55] and **2m** [56], as well as those of ketone **2n** [26], phenylpentane **5** [57], bibenzyl **6** [58] and the byproduct 3-(benzylamino)propane-1,2-diol **7** [58] were assessed by a comparison of their ^1^H spectroscopic data with those reported in the literature. In some cases (see manuscript) ^1^H NMR analysis of the reaction crude showed that the competing reduction had occurred, besides the expected 1,2-nucleophilic addition.

## 4. Conclusions

In the present study, an innovative approach to DES design was used in order to synthesize a novel mixture, namely AGly:ChCl 3:1, whose physico-chemical characterization confirms the possibility of unambiguously classifying it as a deep eutectic solvent. The synthetic usefulness of this DES was demonstrated as the reaction medium in the room-temperature addition of *n*-BuLi to carbonyl compounds under air, affording tertiary alcohols in up to 72% isolated yield. The substrate scope includes acyl chlorides, as yet unexplored in DESs, from which pure tertiary-alcohol products were isolated, together with benzyl chloride, in the first example of an organolithium-promoted aliphatic nucleophilic substitution in a protic medium, under air at room temperature. Chirality transfer from the medium to the chiral products was not observed, but the optical activity of the novel DES offers room for improvement in pursuing this challenging aim.

## Figures and Tables

**Table 1:** Physical and chemical data of AGly:ChCl mixtures.

AGly:ChCl Ratio	AGly (g)	ChCl (g)	Physical State at RT	T_m_ (K) ^1^	h/P	L (mS cm^−1^ M^−1^)
1:1	6.226	9.526	Solid	>353	n.m.	n.m.
2:1	6.008	4.600	Unstable liquid	n.d. (250)	315.41	71 ± 5
3:1	6.144	3.138	Liquid	241 (243)	93.51	22 ± 3
4:1	6.939	2.660	Liquid	246 (248)	11.27	77 ± 7

^1^ The value within the parenthesis is manually measured; n.m. = not measurable; n.d. = not detectable.

**Table 2:** Screening of the reaction conditions for the *n*-BuLi addition to model substrate **1a**.

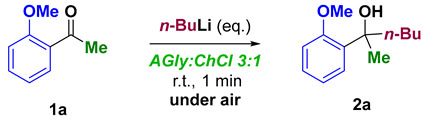			
Entry	DES	*n*-BuLi eq.	Yield 2a (%)
1	Gly:ChCl 2:1	2.0	67
2	AGly:ChCl 3:1	2.0	67
3	AGly:ChCl 3:1	3.0	83
4	AGly	3.0	48 ^1^

^1^ 33% of unreacted **1a** was detected.

**Table 3:** Screening of different DESs as reaction media for the nucleophilic substitution of *n*-BuLi on benzyl chloride **4**.

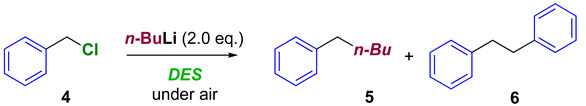			
Entry	DES	Yield 5 (%) ^1^	Yield 6 (%) ^1^
1	Gly:ChCl 2:1	28	3
2	Gly:ChCl 3:1	70	12
3	Urea:ChCl 2:1	61	7
4	AGly:ChCl 3:1	85	11
5	LA:ChCl 2:1	32	2
6	Gly:KF 6:1	74	7
7	H_2_O:ChCl 2:1	-	-
8	EG:ChCl 2:1	34	3

^1^ NMR yield.

## Data Availability

The data reported in this article can be obtained from the authors upon reasonable request.

## Supplementary Materials

The following supporting information can be downloaded at: https://www.mdpi.com/article/10.3390/molecules27238566/s1, 1. DSC profile for AGly:ChCl mixtures in 3:1 and 4:1 ratio. Figure S1. 2. Addition of *n*-butyllithium to carbonyl compounds in DESs; **1a**. Comparison between DESs Gly:ChCl 2:1 and AGly:ChCl 3:1. Figures S2–S5; **1b**. Substrate scope: aromatic ketones. Figures S6–S16; **1c**. Substrate scope: aliphatic ketones. Figures S16–S22; **1d**. Substrate scope: carboxylic acid derivatives (esters, acyl chlorides, amides). Figures S23–S28; 2. Addition of *n*-butyllithium to benzyl chloride in DESs. Figures S29–S36.

## Sample Availability

Samples of the compounds **2** are available from the authors.
