# Supplementary material for: Design of a New Chiral Deep Eutectic Solvent Based on 3-Amino-1,2-propanediol and Its Application in Organolithium Chemistry
Source: Molecules. 2022 Dec 5;27(23):8566. doi: 10.3390/molecules27238566 (PMC9738533; doi:10.3390/molecules27238566)
Supplement: Supplementary file 1 [file molecules-27-08566-s001.zip › molecules-2043350-supplementary.pdf]

Supplementary Material

for

Design of a new chiral Deep Eutectic Solvent based on 3-Amino-1,2-propanediol and its application in organolithium chemistry

Achille Antenucci [1,2*], Matteo Bonomo [1,3*], Simone Ghinato [1], Marco Blangetti [1] and Stefano Dughera [1*]

[1] Department of Chemistry, Università degli Studi di Torino, via Pietro Giuria 7, 10125 Torino, Italy.

[2] Centro Ricerche per la Chimica Fine s.r.l. for Silvateam s.p.a., via Torre 7, 12080 San Michele Mondovì (CN), Italy

[3] NIS Interdepartmental Centre and INSTM Reference Centre, Università degli Studi di Torino, via Gioacchino Quarello 15/a, 10125 Torino, Italy

AA: achille.antenucci@unito.it; MB: matteo.bonomo@unito.it; SD: stefano.dughera@unito.it

1. **DSC profile for AGly:ChCl mixtures in 3:1 and 4:1 ratio; pag. 2**

2. **Addition of *n*-butyllithium to carbonyl compounds in DESs; pag. 2**

   2a. General consideration on NMR yield; pag. 2
   2b. Comparison between DESs Gly:ChCl 2:1 and AGly:ChCl 3:1; pag. 3
   2c. Substrate scope: aromatic ketones; pag. 5
   2d. Substrate scope: aliphatic ketones; pag. 11
   2e. Substrate scope: carboxylic acid derivatives (esters, acyl chlorides, amides); pag. 14

3. **Addition of *n*-butyllithium and phenyllithium to benzyl chloride in DESs; pag. 17**

4. **Bigliographic references, pag. 25**

1. DSC profile for AGly:ChCl mixtures in 3:1 and 4:1 ratio

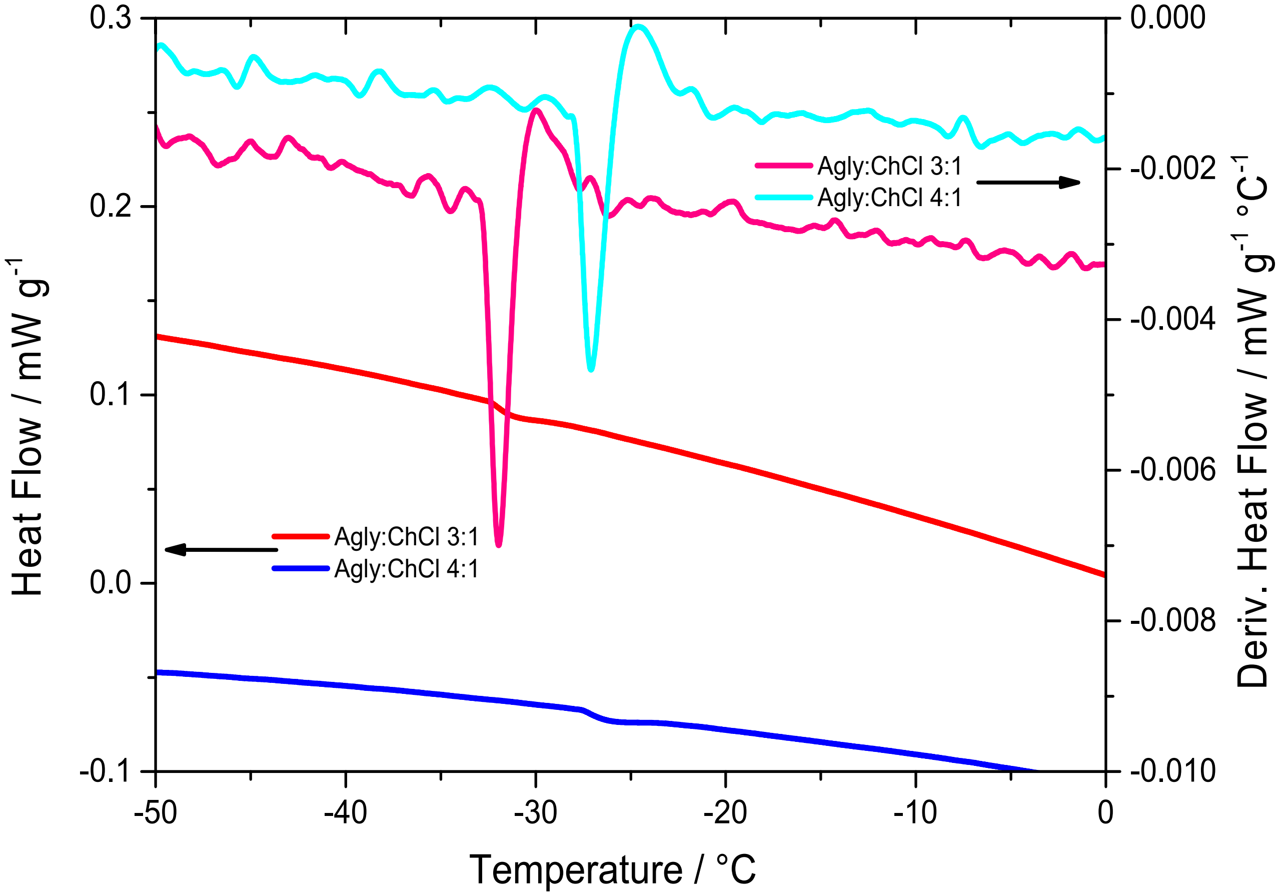

Figure S1 DSC profile for AGly:ChCl mixtures in 3:1 and 4:1 ratio

2. Addition of *n*-butyllithium to carbonyl compounds in DESs

2a. General consideration on NMR yield

Quantification on reaction not affording complete conversion of the substrate was performed with nitromethane as an internal standard and the NMR yield has been determined by Equation S1:

Equation S1 $$\text{yield (\%)} = \frac{I \times n_{CH3NO2}}{n_{substrate}} \times f \times 100$$ Where:

I = normalized value of integral (value of integral/number of protons)

n_{CH3NO2} = mmol of internal standard (nitromethane)

$n_{substrate}$ = mmol of substrate

f = dilution factor

2b. Comparison between DESs Gly:ChCl 2:1 and AGly:ChCl 3:1

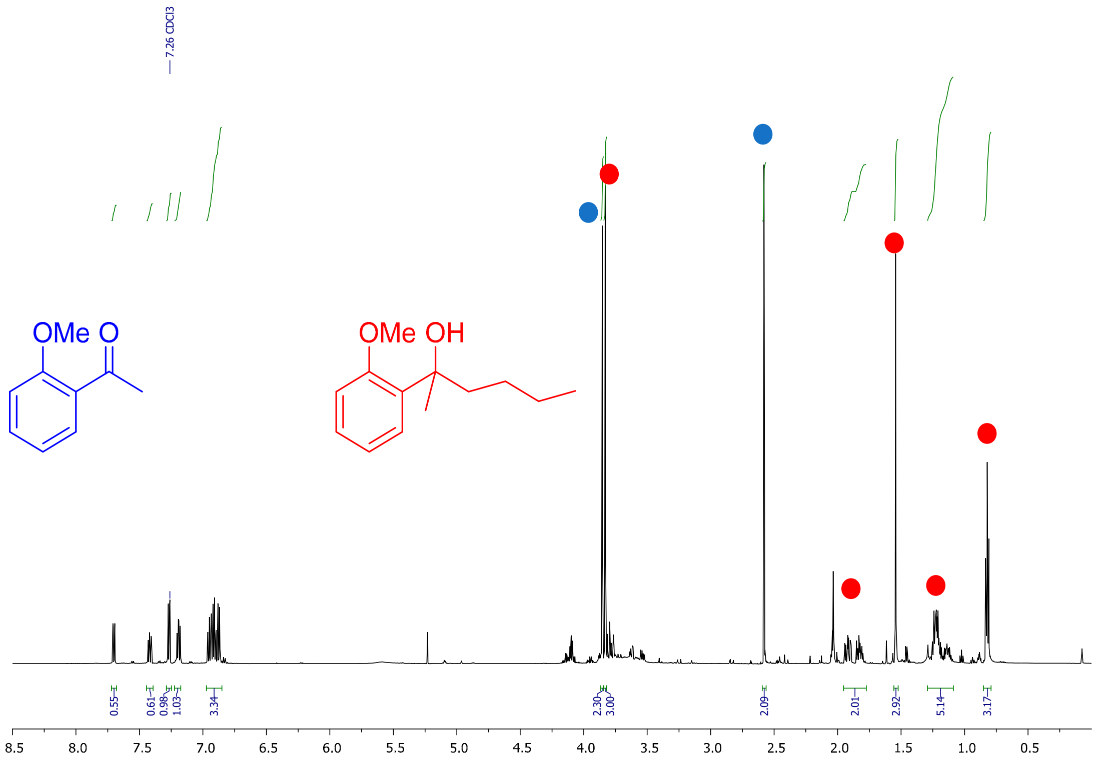

Figure S2 ^{1}H NMR spectrum ($CDCl_3$) of the reaction crude (254 mg) of the addition of 1.96 M *n*-BuLi (2.0 eq.) to 2'-methoxyacetophenone (**1a**) in Gly:ChCl 2:1.

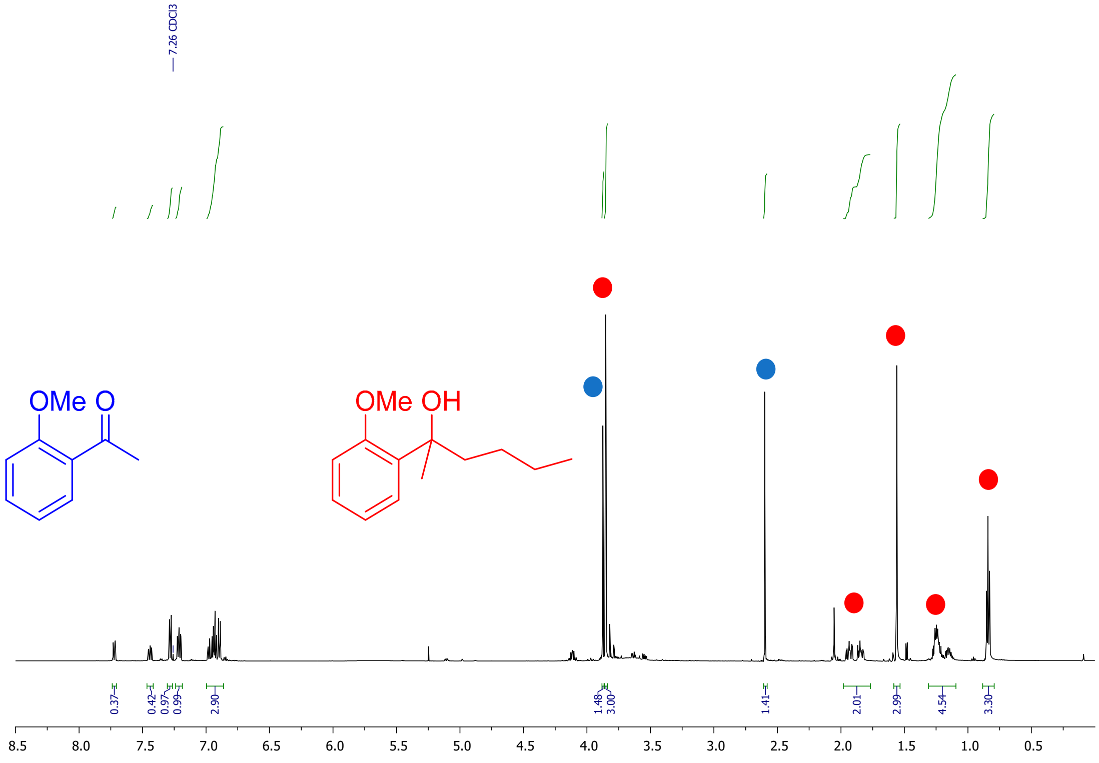

Figure S3 ^{1}H NMR spectrum ($CDCl_3$) of the reaction crude (199 mg) of the addition of 2.08 M *n*-BuLi (2.0 eq.) to 2'-methoxyacetophenone (**1a**) in Gly:ChCl 2:1.

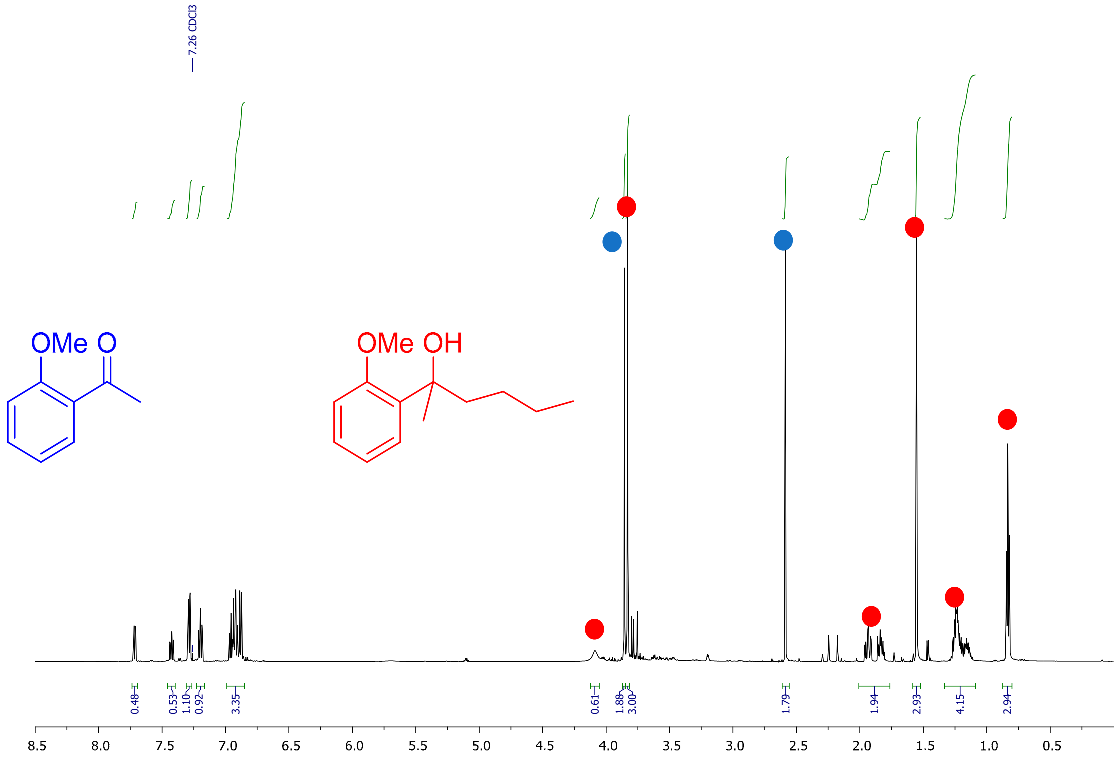

Figure S4 ^{1}H NMR spectrum ($CDCl_3$) of the reaction crude (230 mg) of the addition of 1.96 M *n*-BuLi (2.0 eq.) to 2'-methoxyacetophenone (**1a**) in AGly:ChCl 3:1.

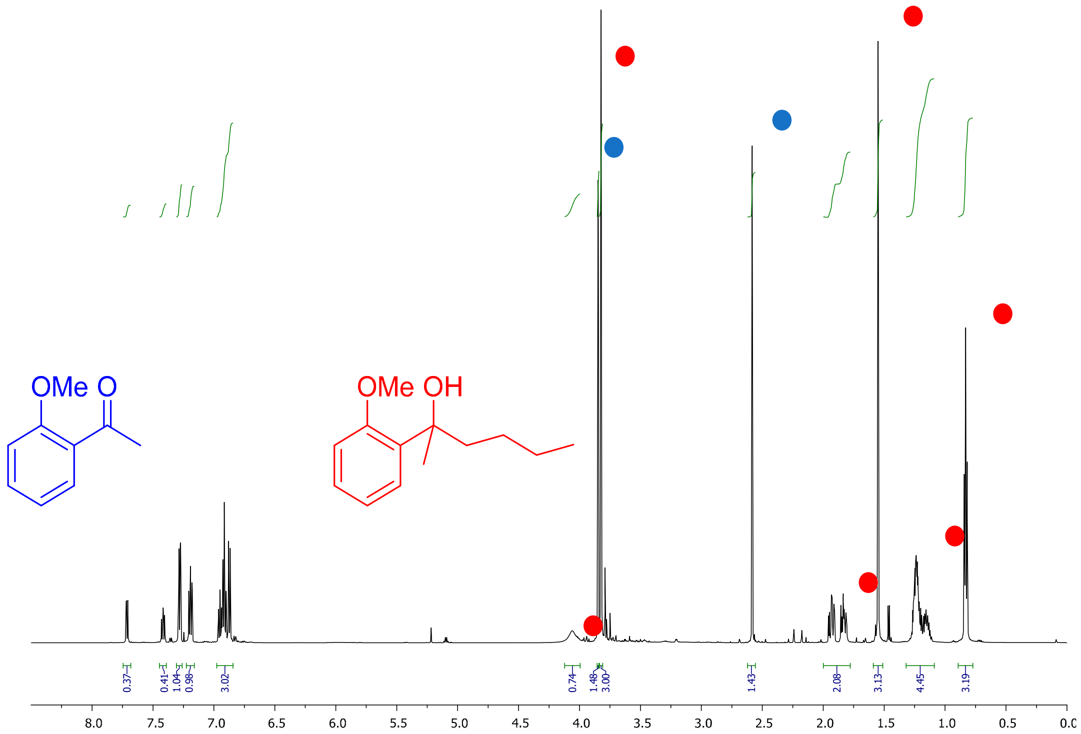

Figure S5 ^{1}H NMR spectrum ($CDCl_3$) of the reaction crude (253 mg) of the addition of 2.08 M *n*-BuLi (2.0 eq.) to 2'-methoxyacetophenone (**1a**) in AGly:ChCl 3:1.

2c. Substrate scope: aromatic ketones

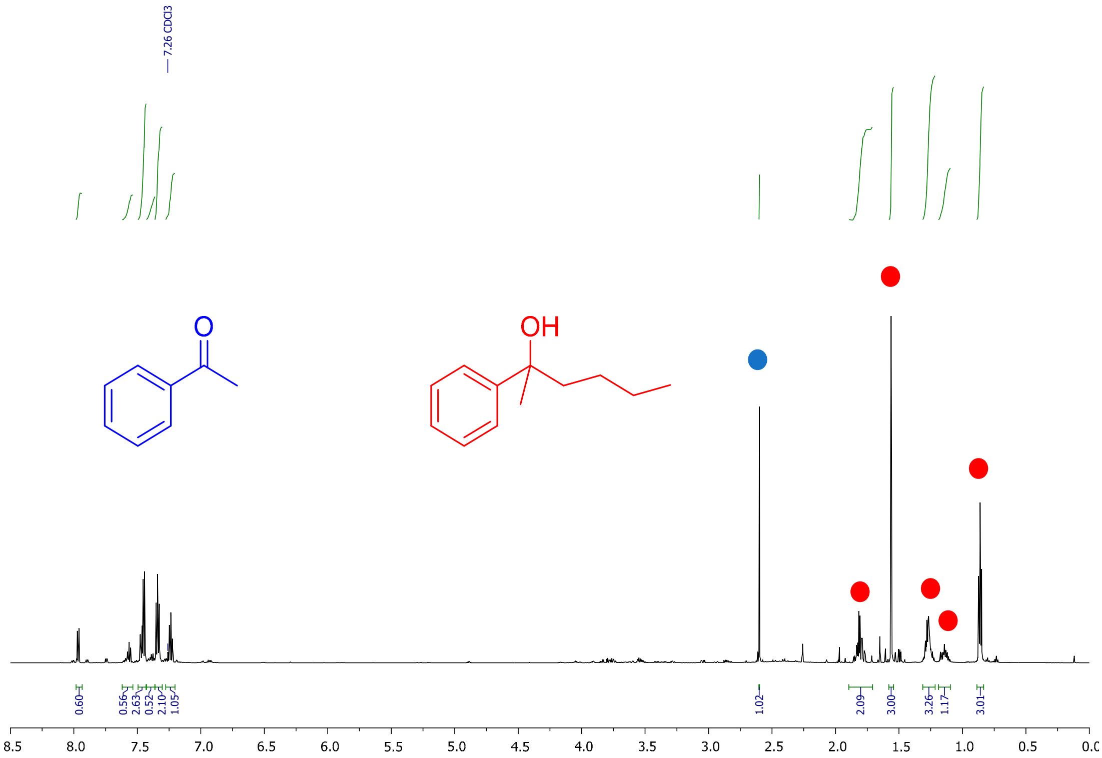

Figure S6 ^{1}H NMR spectrum ($CDCl_3$) of the reaction crude (180 mg) of the addition of 2.08 M *n*-BuLi (2.0 eq.) to acetophenone (**1b**) in AGly:ChCl 3:1.

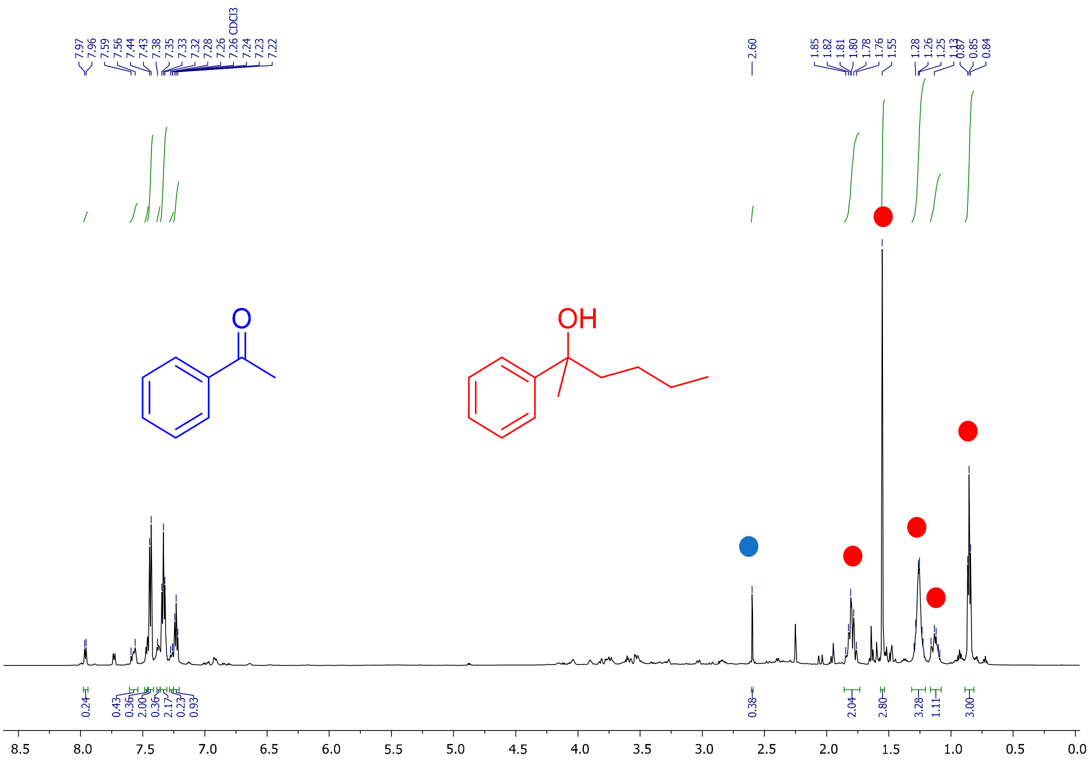

Figure S7 ^{1}H NMR spectrum ($CDCl_3$) of the reaction crude (155 mg) of the addition of 2.5 M *n*-BuLi (3.0 eq.) to acetophenone (**1b**) in AGly:ChCl 3:1.

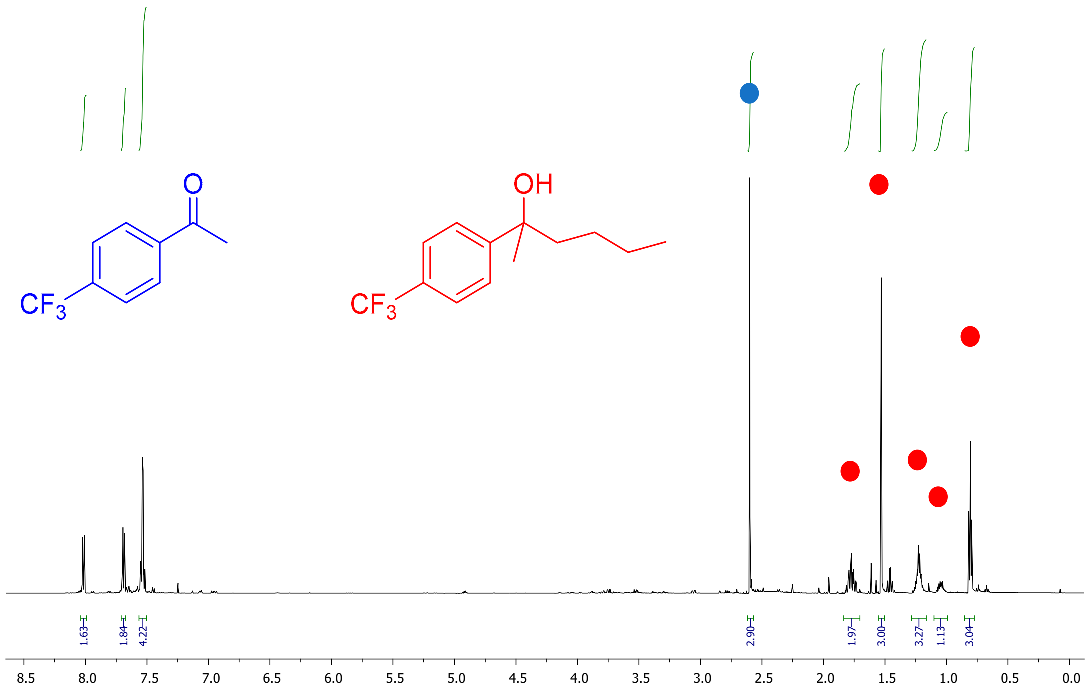

Figure S8 ^{1}H NMR spectrum ($CDCl_3$) of the reaction crude (285 mg) of the addition of 2.08 M *n*-BuLi (2.0 eq.) to 4’-trifluoromethylacetophenone (**1c**) in AGly:ChCl 3:1.

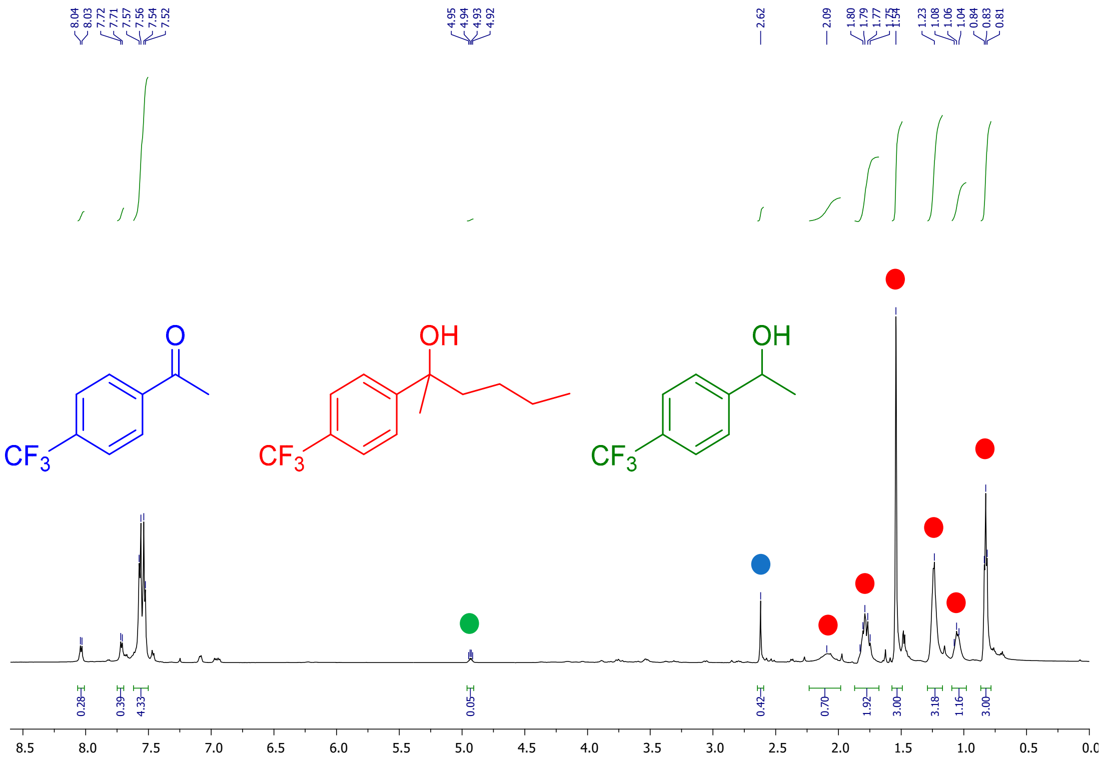

Figure S9 ^{1}H NMR spectrum ($CDCl_3$) of the reaction crude (274 mg) of the addition of 2.5 M *n*-BuLi (3.0 eq.) to 4’-trifluoromethylacetophenone (**1c**) in AGly:ChCl 3:1.

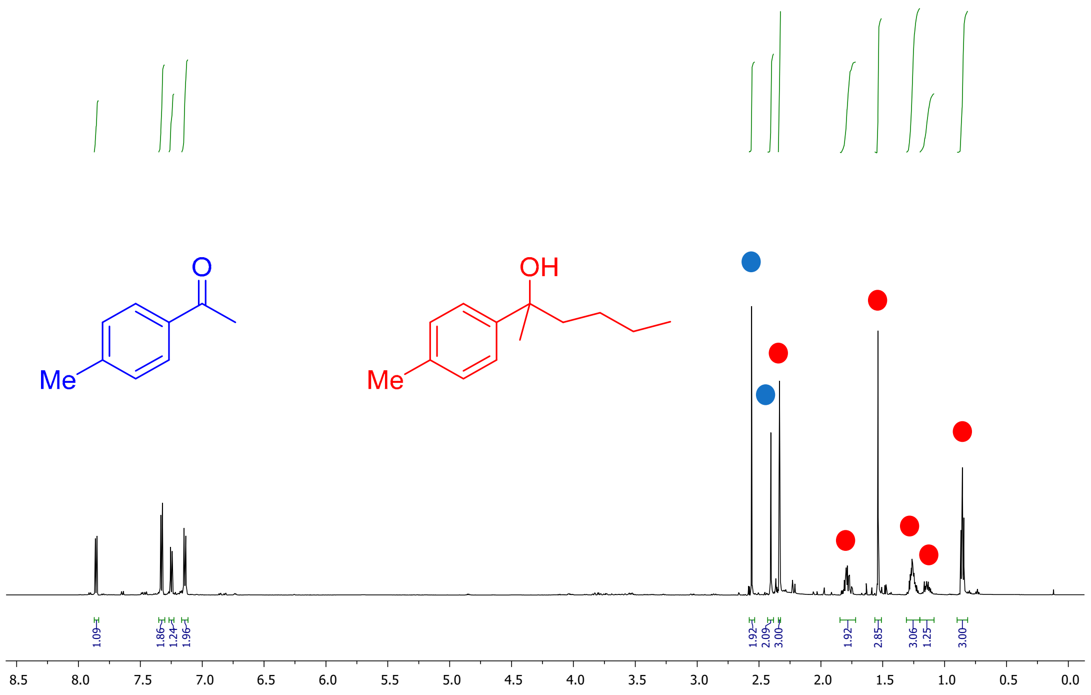

Figure S10 ^{1}H NMR spectrum ($CDCl_3$) of the reaction crude (196 mg) of the addition of 2.08 M *n*-BuLi (2.0 eq.) to 4'-methylacetophenone (**1d**) in AGly:ChCl 3:1.

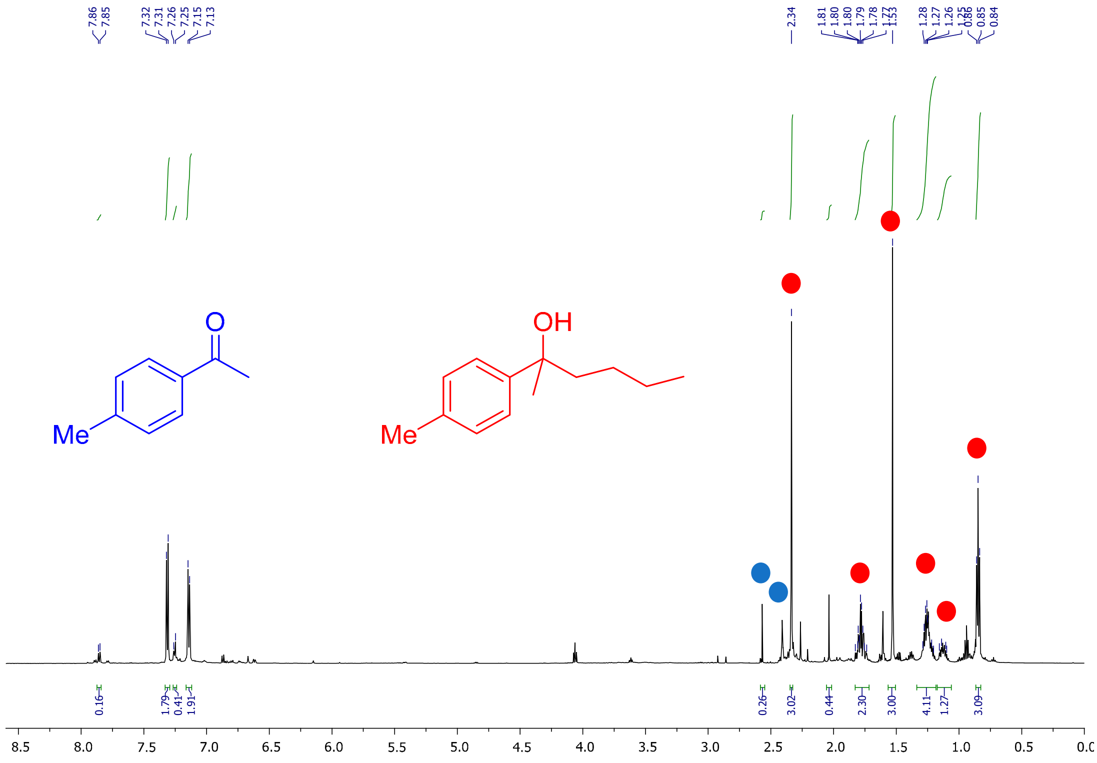

Figure S11 ^{1}H NMR spectrum ($CDCl_3$) of the reaction crude (183 mg) of the addition of 2.5 M *n*-BuLi (3.0 eq.) to 4'-methylacetophenone (**1d**) in AGly:ChCl 3:1.

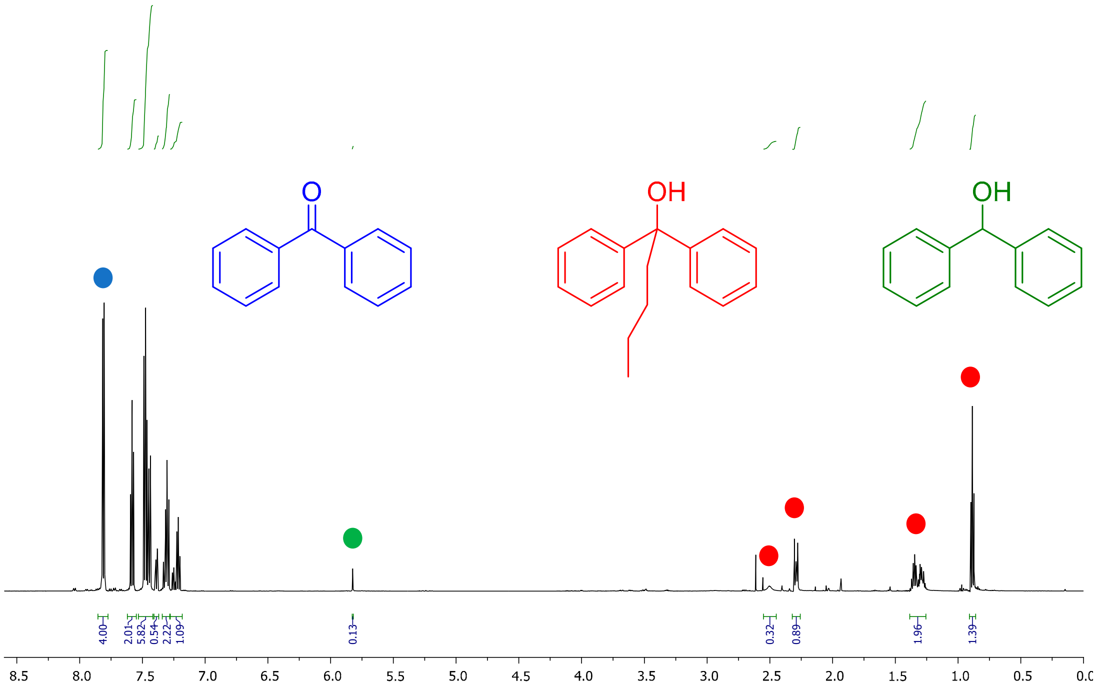

Figure S12 ^{1}H NMR spectrum ($CDCl_3$) of the reaction crude (192 mg) of the addition of 2.08 M *n*-BuLi (2.0 eq.) to benzophenone (**1e**) in AGly:ChCl 3:1.

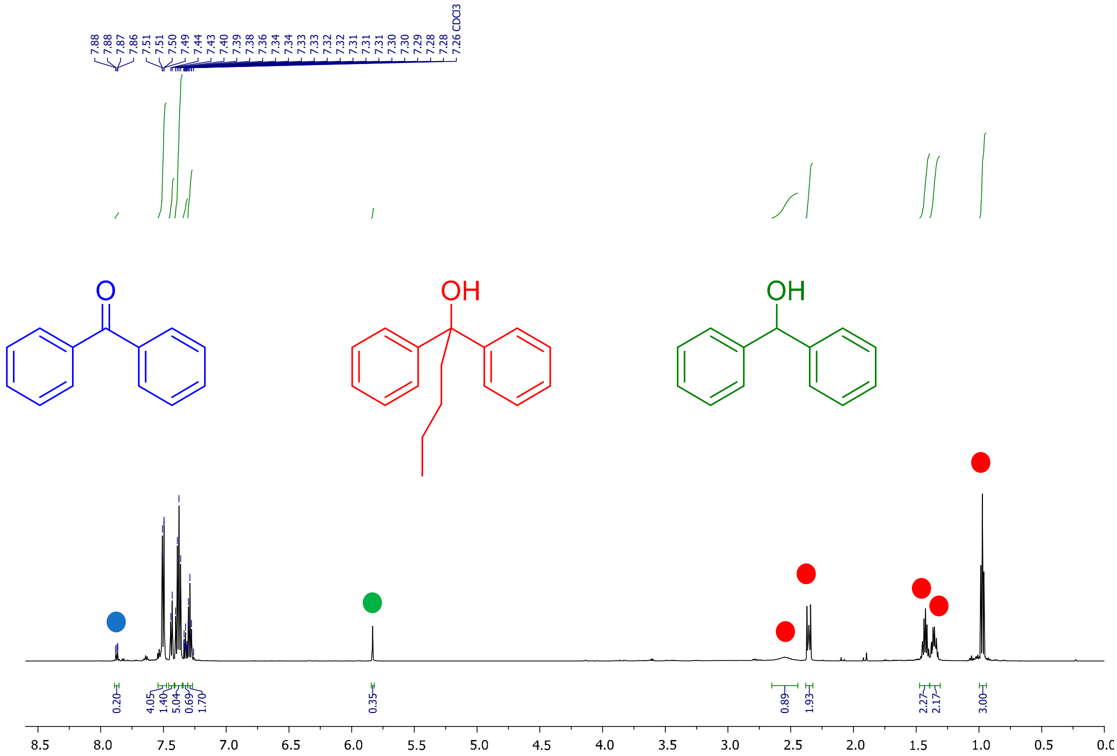

Figure S13 ^{1}H NMR spectrum ($CDCl_3$) of the reaction crude (222 mg) of the addition of 2.08 M *n*-BuLi (3.0 eq.) to benzophenone (**1e**) in AGly:ChCl 3:1.

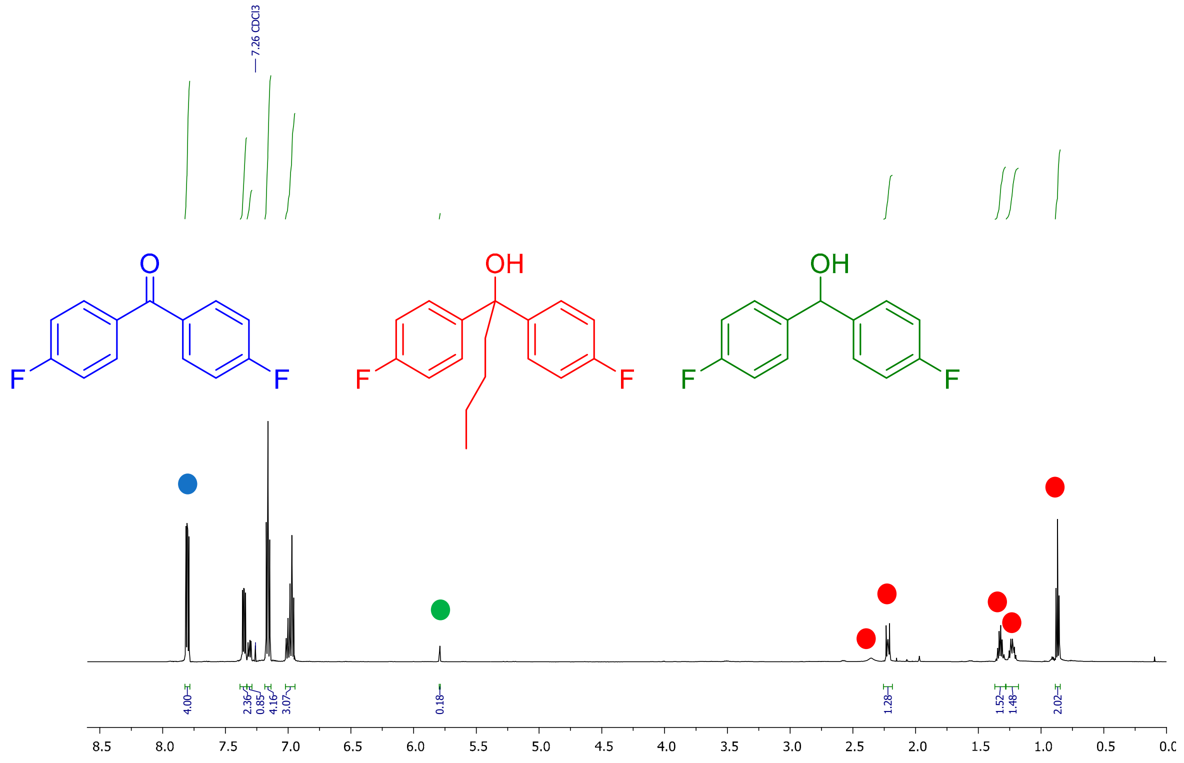

Figure S14 ^{1}H NMR spectrum ($CDCl_3$) of the reaction crude (203 mg) of the addition of 2.08 M *n*-BuLi (2.0 eq.) to 4,4'-difluorobenzophenone (**1f**) in AGly:ChCl 3:1.

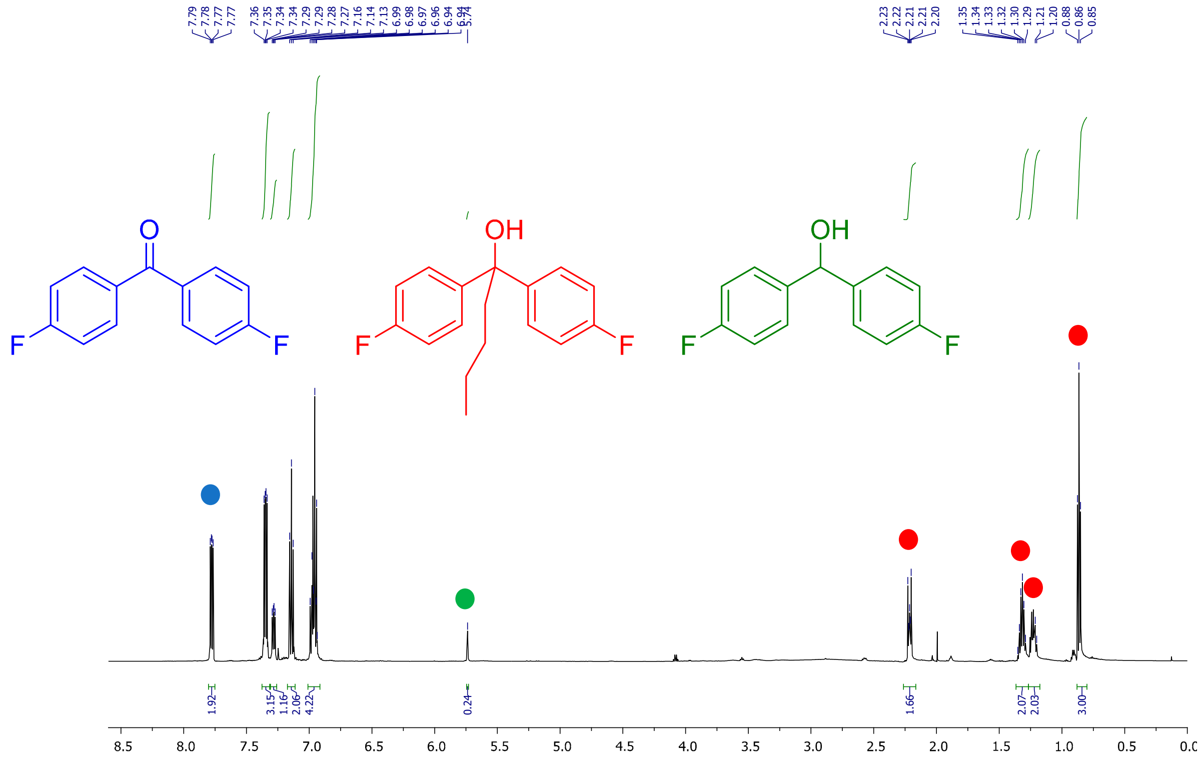

Figure S15 ^{1}H NMR spectrum ($CDCl_3$) of the reaction crude (237 mg) of the addition of 2.08 M *n*-BuLi (3.0 eq.) to 4,4'-difluorobenzophenone (**1f**) in AGly:ChCl 3:1.

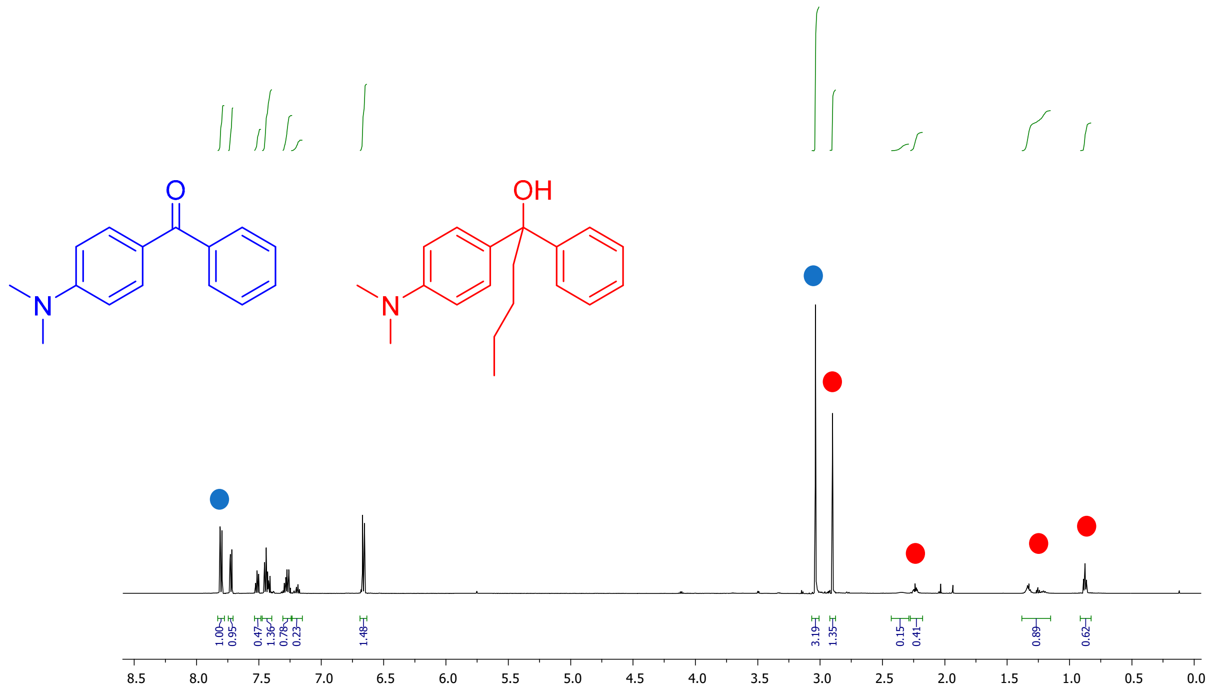

Figure S16 ^{1}H NMR spectrum ($CDCl_3$) of the reaction crude (232 mg) of the addition of 2.08 M *n*-BuLi (2.0 eq.) to 4-dimethylaminobenzophenone (**1g**) in AGly:ChCl 3:1.

2d. Substrate scope: aliphatic ketones

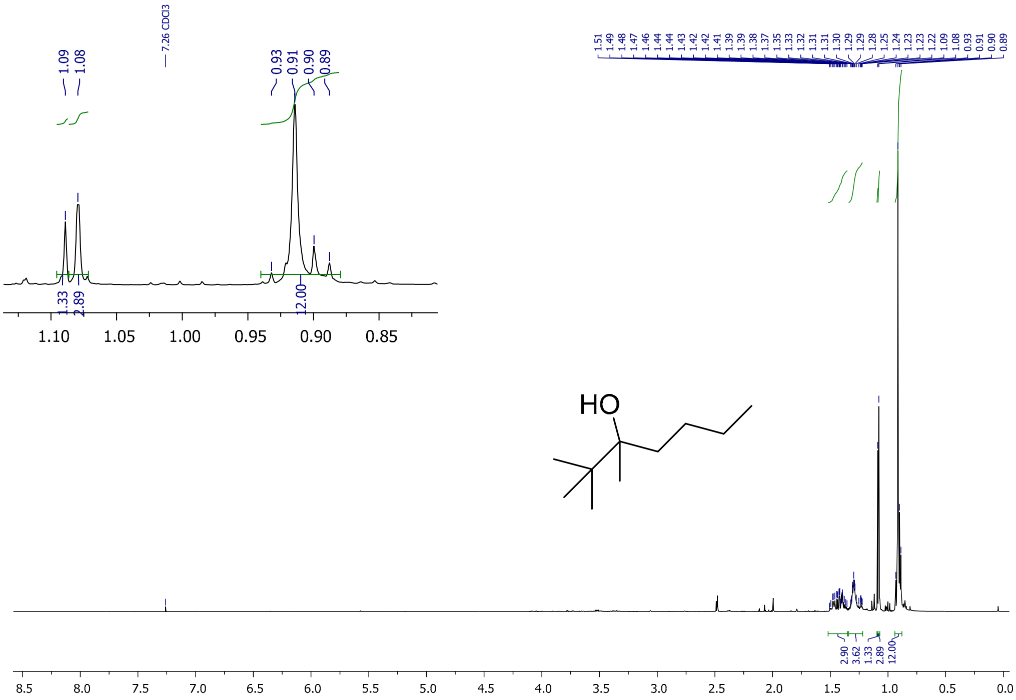

Figure S17: ^{1}H NMR of 2h (600 MHz, $CDCl_3$) δ 1.51 – 1.35 (m, 3H), 1.34 – 1.21 (m, 4H), 1.08 (s, 3H), 0.95 – 0.88 (m, 12H). Isolated mass = 88 mg

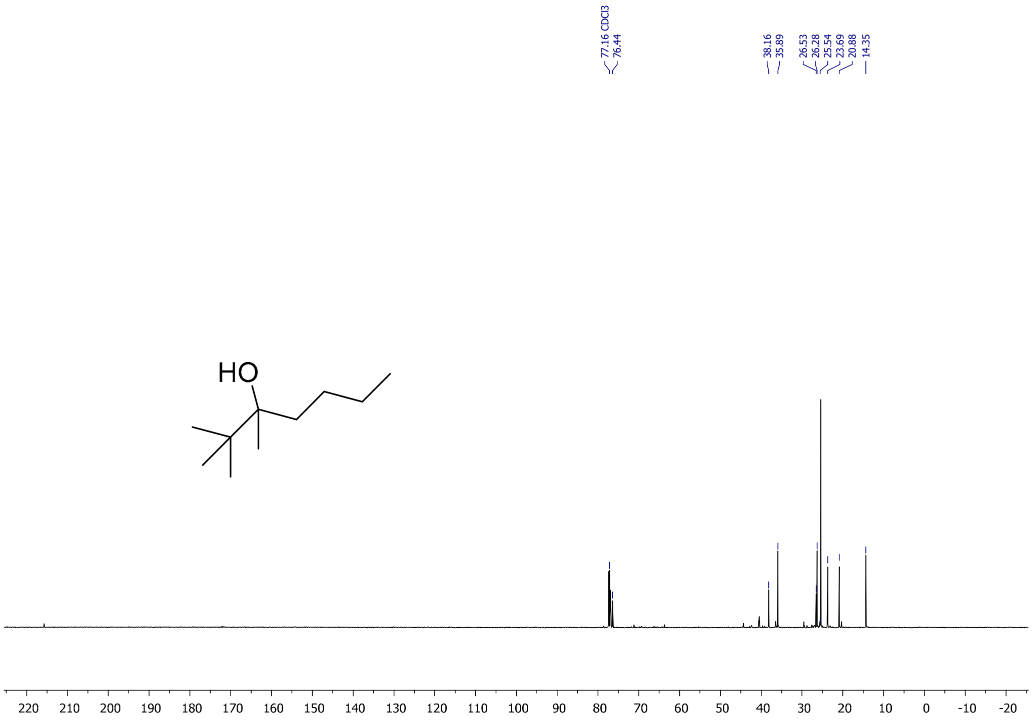

Figure S18: ^{13}C NMR of 2h (151 MHz, CDCl3) δ 76.44, 38.16, 35.89, 26.53, 26.28, 25.54, 23.69, 20.88, 14.35. Isolated mass = 88 mg

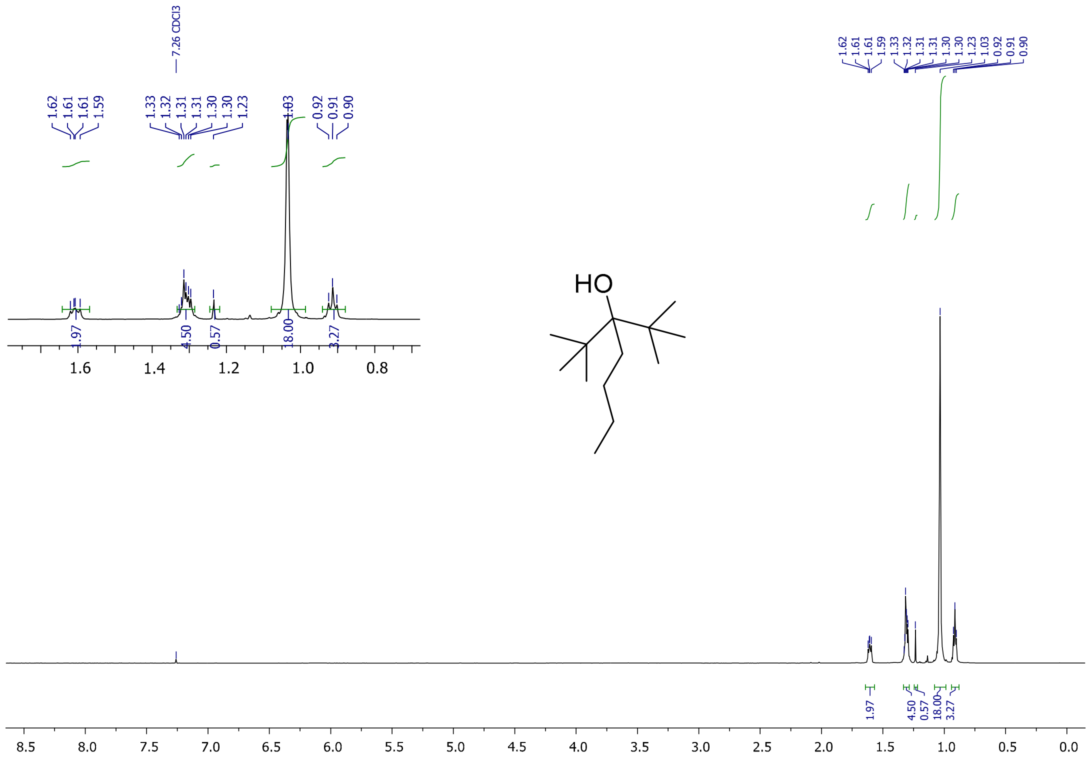

Figure S19: ^{1}H NMR of 2i (600 MHz, $CDCl_3$) δ 1.61 (m, 2H), 1.31 (m, 4H), 1.23 (br s, 1H), 1.03 (s, 18H), 0.91 (t, *J* = 6.7 Hz, 3H). Isolated mass = 145 mg

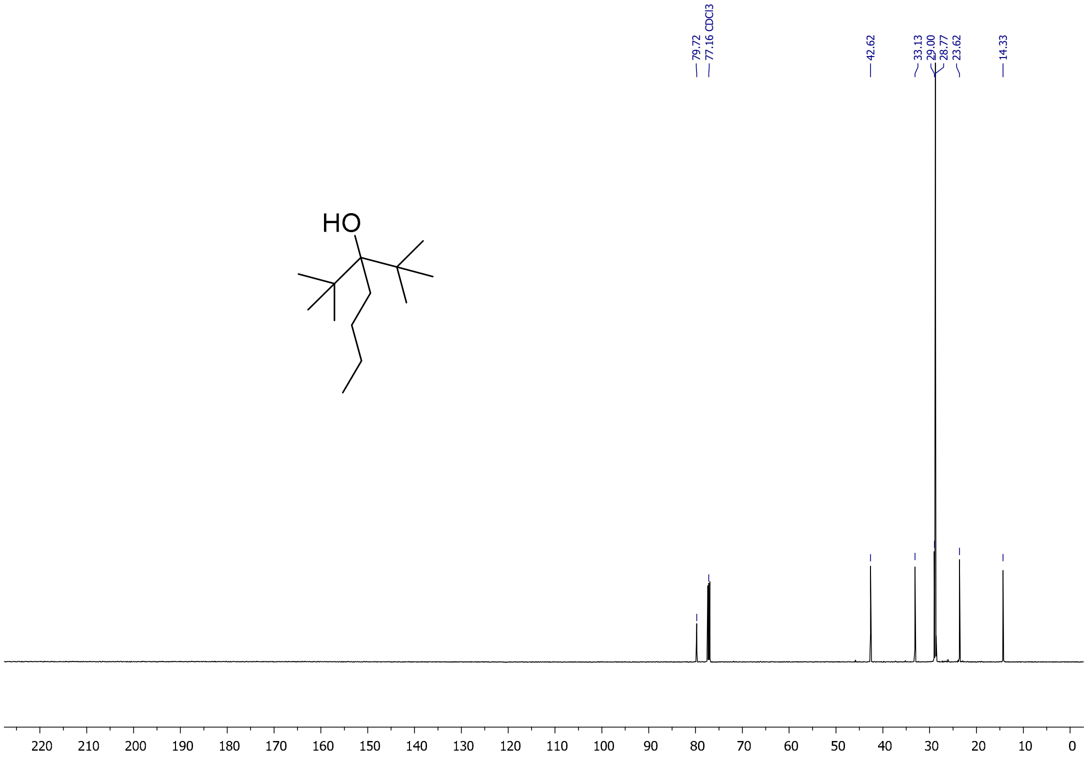

Figure S20: ^{13}C NMR of 2i (151 MHz, $CDCl_3$) δ 79.72, 42.62, 33.13, 29.00, 28.77, 23.62, 14.33.

Isolated mass = 145 mg

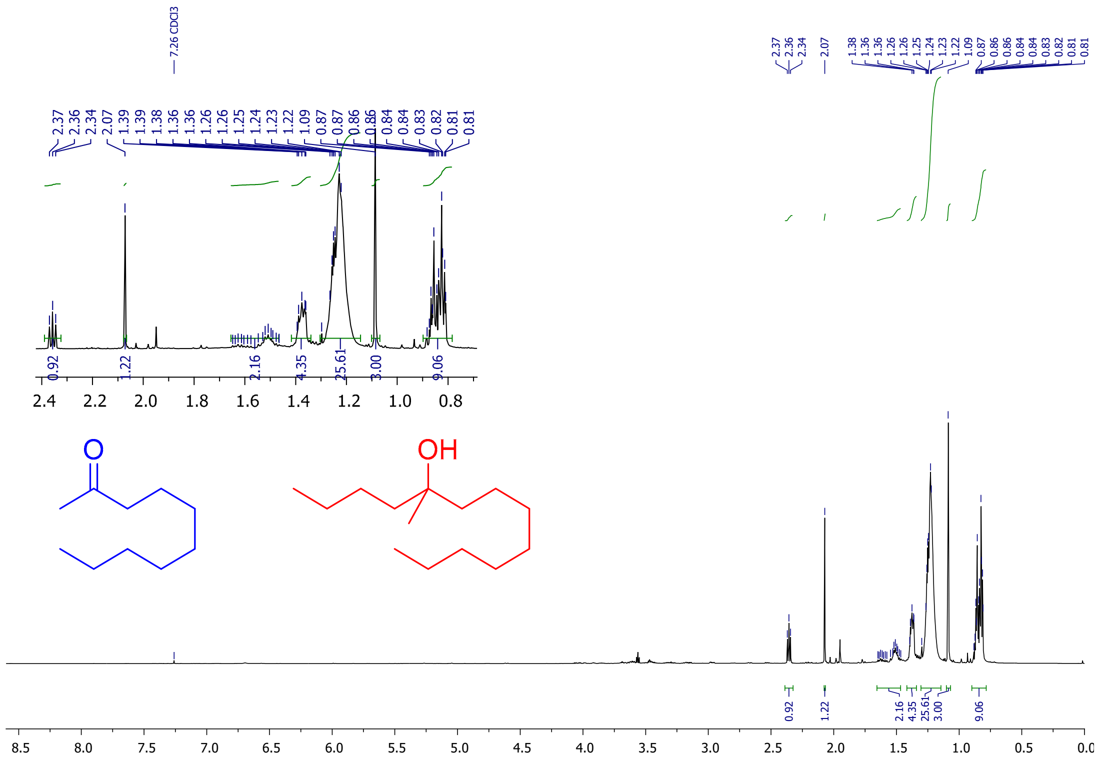

Figure S21 ^{1}H NMR spectrum ($CDCl_3$) of the reaction crude (184 mg) of the addition of 2.08 M *n*-BuLi (2.0 eq.) to 2-decanone (**1j**) in AGly:ChCl 3:1.

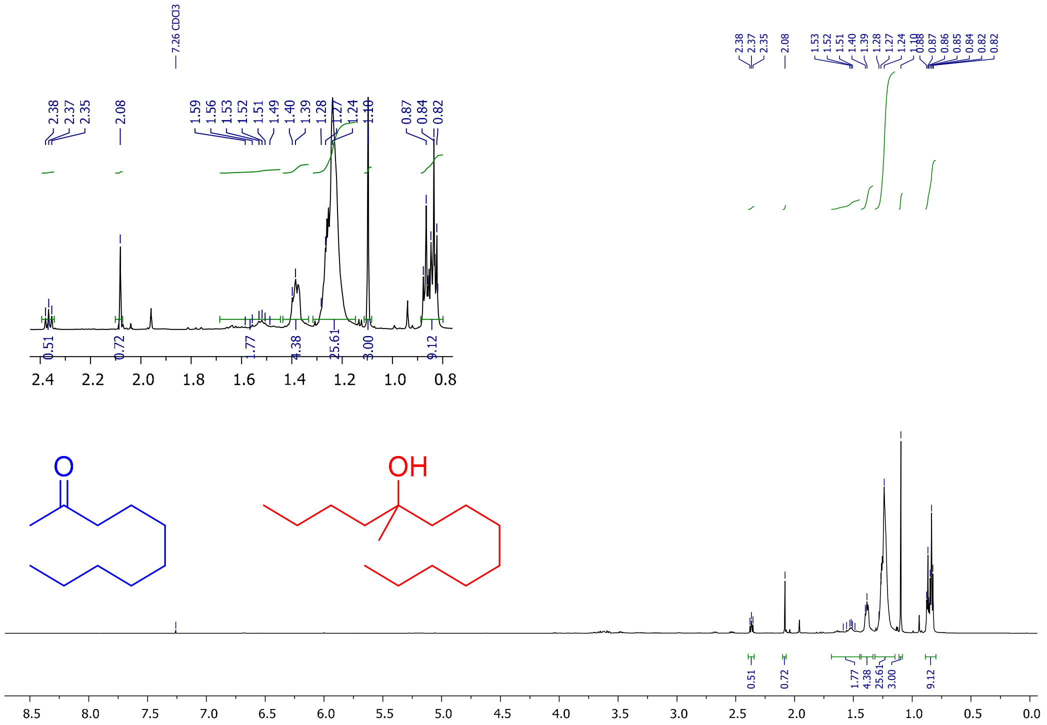

Figure S22 ^{1}H NMR spectrum ($CDCl_3$) of the reaction crude (190 mg) of the addition of 2.08 M *n*-BuLi (3.0 eq.) to 2-decanone (**1j**) in AGly:ChCl 3:1.

2e. Substrate scope: carboxylic acid derivatives (esters, acyl chlorides, amides)

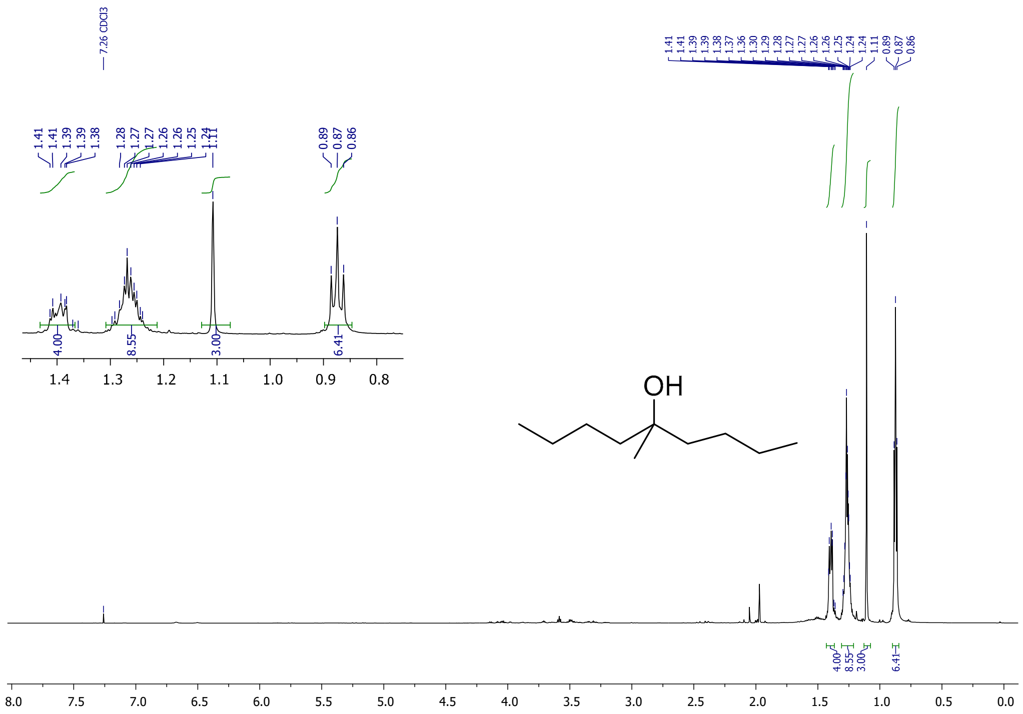

Figure S23 ^{1}H NMR of 2n (600 MHz, CDCl$_3$) δ 1.41 – 1.36 (m, 4H), 1.30 – 1.24 (m, 9H), 1.11 (s, 3H), 0.87 (t, *J* = 7.0 Hz, 6H). Isolated mass = 98 mg (from **1n**); 16 mg (from **1o**)

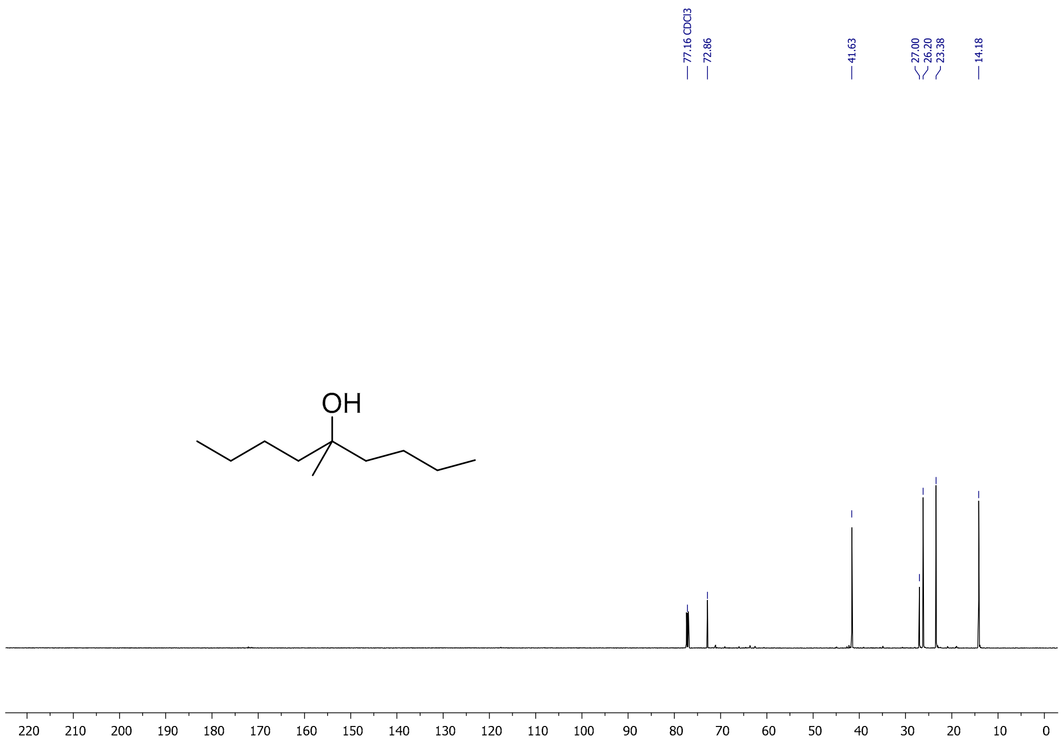

Figure S24 ^{13}C NMR of 2n (151 MHz, CDCl$_3$) δ 72.86, 41.63, 27.00, 26.20, 23.38, 14.18.

Isolated mass = 98 mg (from **1n**); 16 mg (from **1o**)

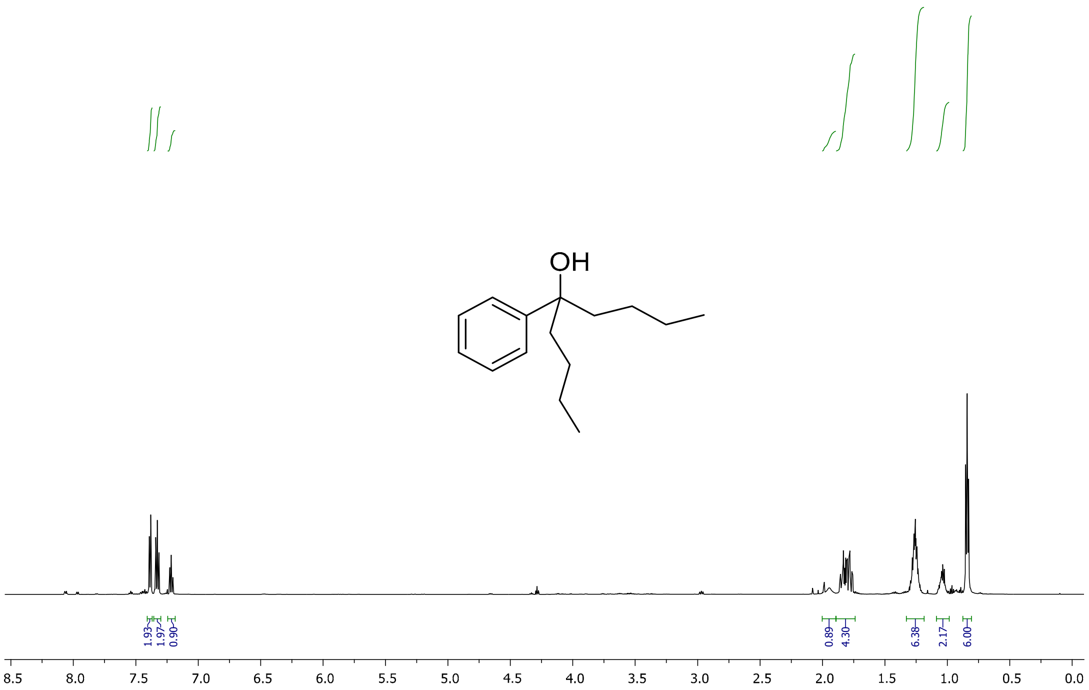

Figure S25 ^{1}H NMR of 2l (600 MHz, $CDCl_3$) δ 7.39 (dd, *J* = 8.4, 1.2 Hz, 2H), 7.33 (t, *J* = 7.8 Hz, 2H), 7.22 (tt, J = 7.5, 1.2 Hz, 1H), 1.95 (br s, 1H), 1.89 – 1.74 (m, 4H), 1.31 – 1.20 (m, 6 H), 1.09 – 0.98 (m, 2H), 0.84 (t, *J* = 7.3 Hz, 6H). Isolated mass = 143 mg (from **1l**); 108 mg (from **1m**)

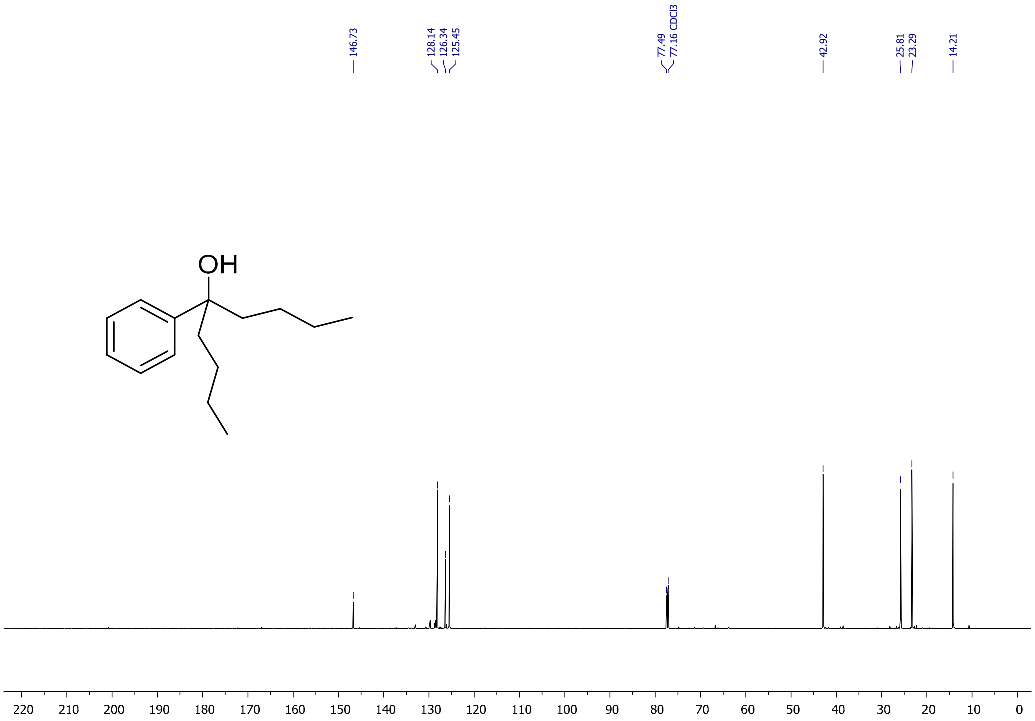

Figure S26 ^{13}C NMR of 2l (151 MHz, $CDCl_3$) δ 146.73, 128.14, 126.34, 125.45, 77.49, 42.92, 25.81, 23.29, 14.21. Isolated mass = 143 mg (from **1l**); 108 mg (from **1m**)

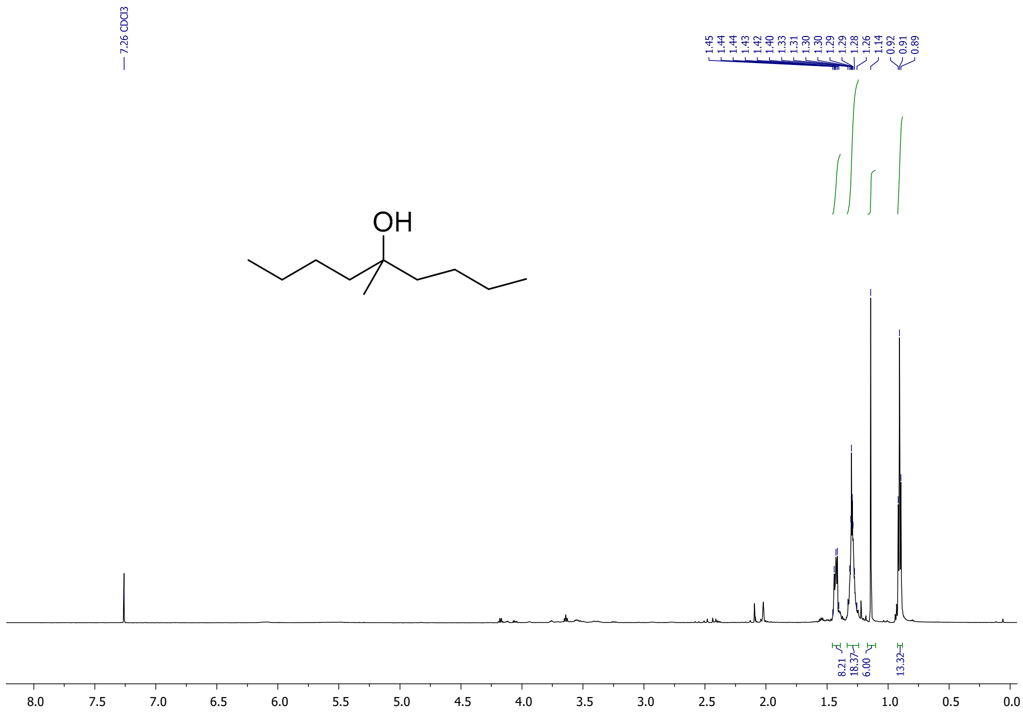

Figure S27 ^{1}H NMR spectrum ($CDCl_3$) of the reaction crude (16 mg) of the addition of 2.08 M *n*-BuLi (3.0 eq.) to acetyl chloride (**1m**) in AGly:ChCl 3:1.

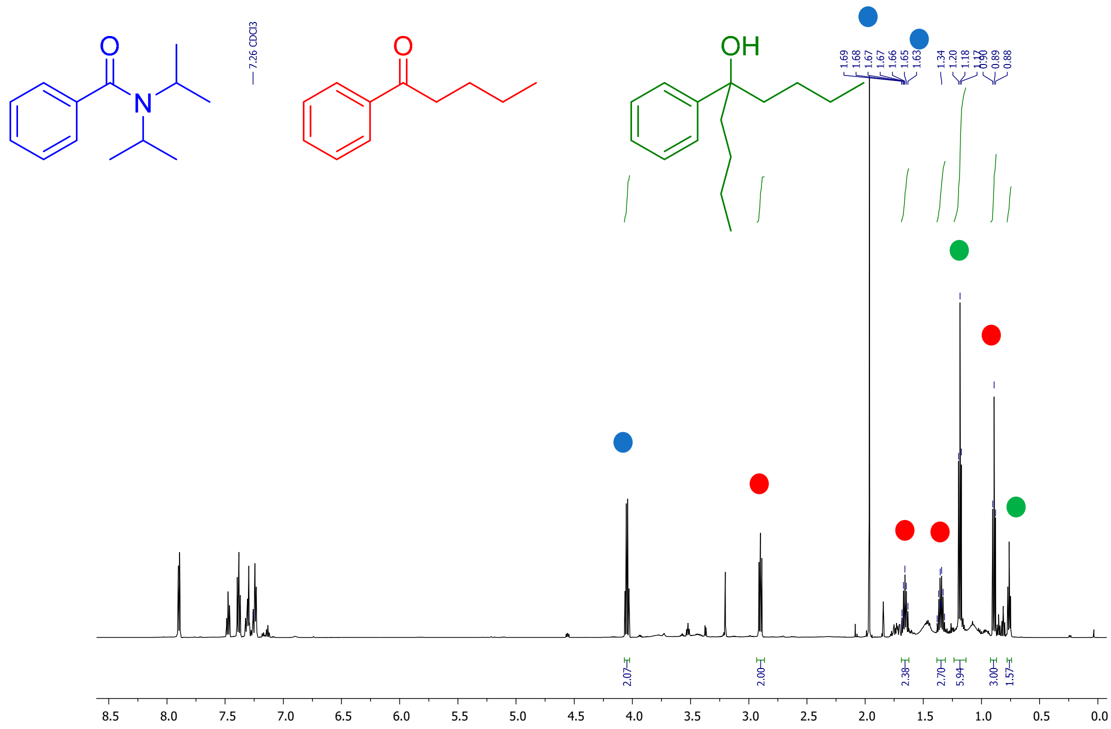

Figure S28 ^{1}H NMR spectrum ($CDCl_3$) of the reaction crude (247 mg) of the addition of 2.08 M *n*-BuLi (2.0 eq.) to N,N-diisopropylbenzamide (**1p**) in 5 g of AGly:ChCl 3:1 and 1 mL CPME at 0 °C.

3. Addition of *n*-butyllithium to benzyl chloride in DESs

Figure S29: (2.0 eq. *n*-BuLi 2.5 M, 20 min, AGly:ChCl 3:1)

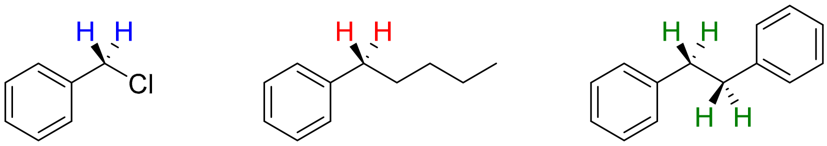

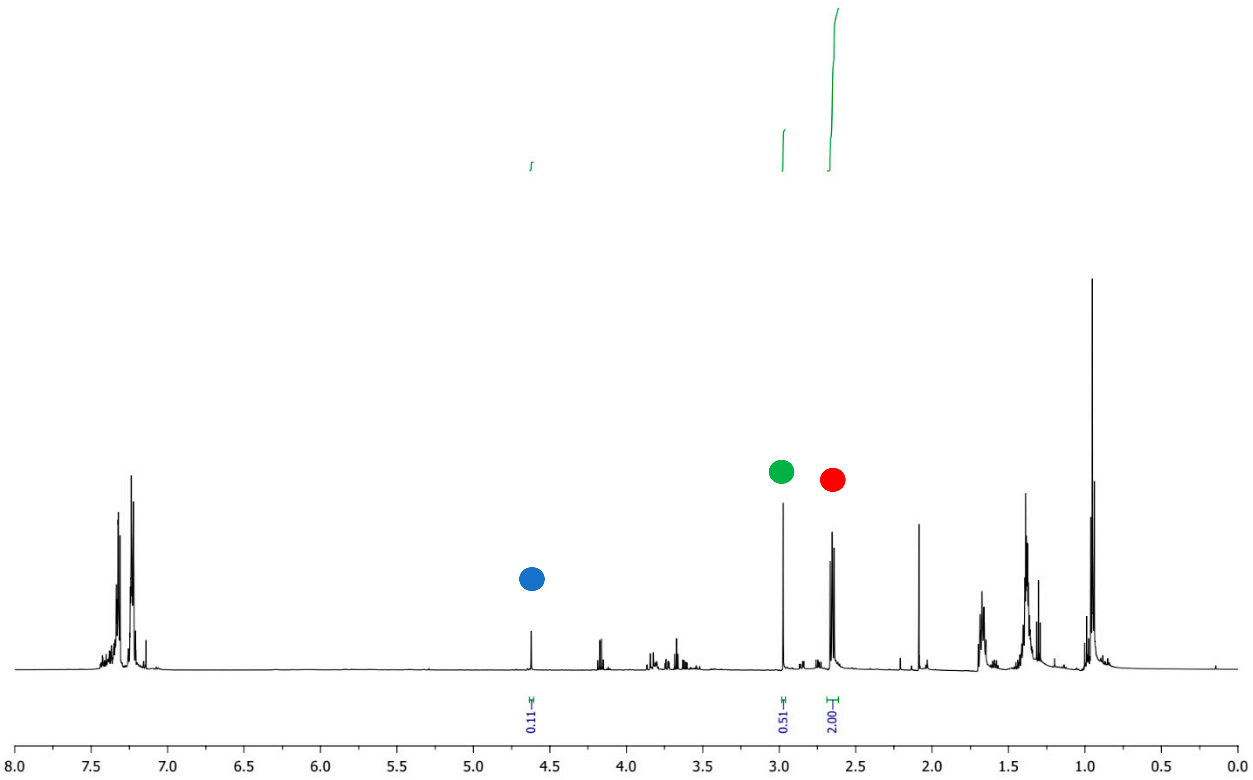

0.11 : **0.255** : **2.00**

4.65%, **84.57%**, **10.78%**

Isolated mass = 130 mg

Figure S30 (2.0 eq. *n*-BuLi 2.5 M, 20 min, Urea:ChCl 2:1)

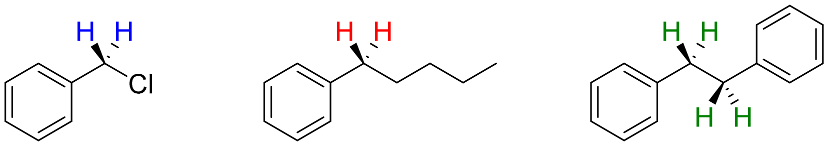

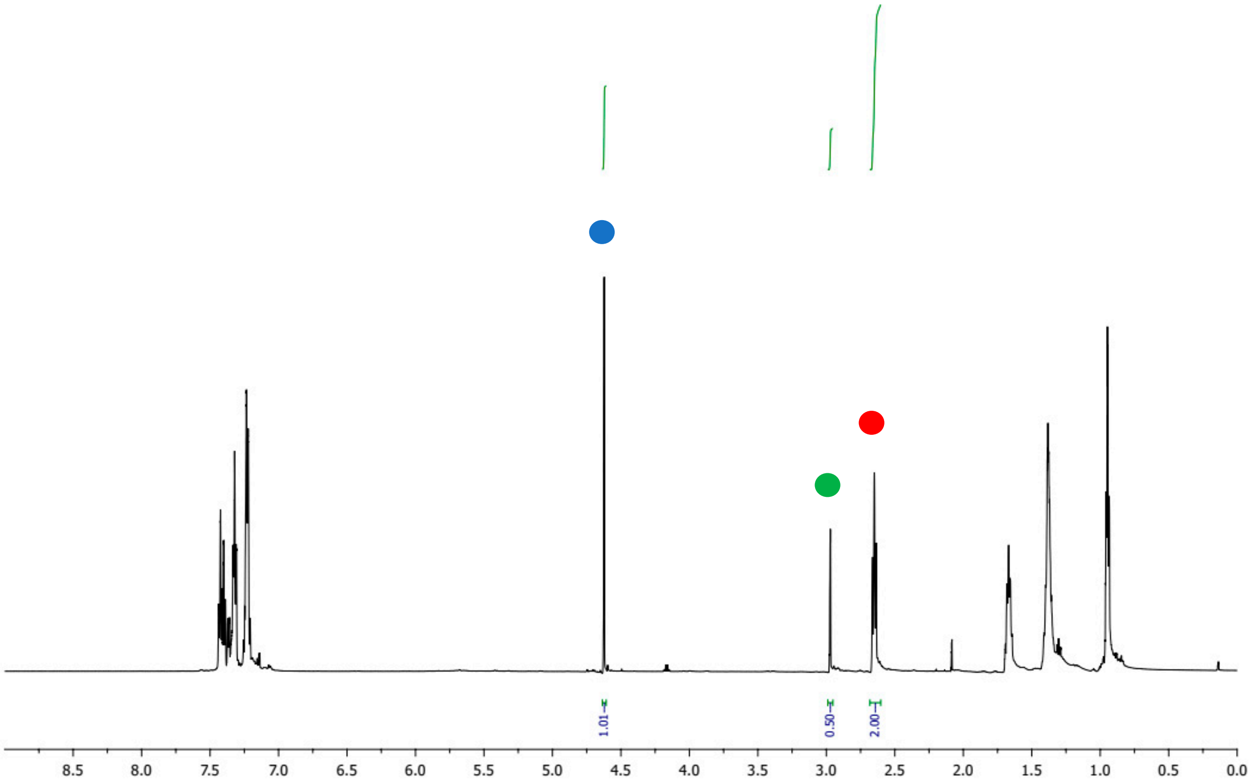

1.01 : 0.25 : 2.00

30.98%, 61.35%, 6.67%

Isolated mass = 84 mg

Figure S31 (2.0 eq. *n*-BuLi 2.5 M, 20 min, LA:ChCl 2:1)

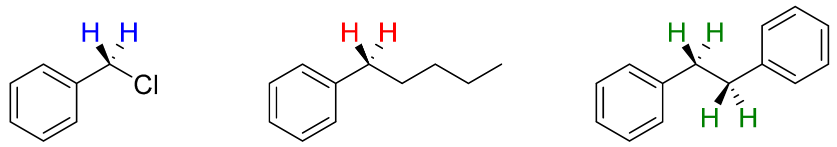

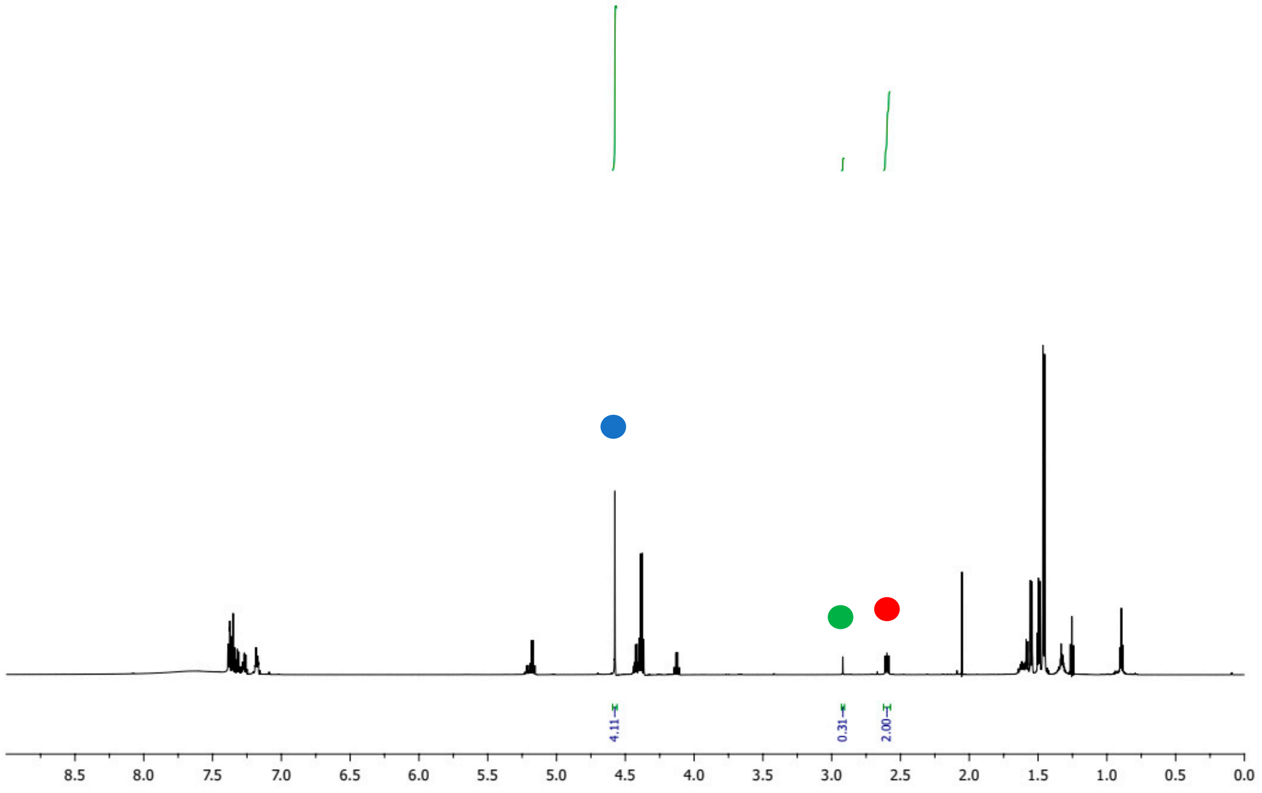

4.11 : 0.155 : 2.00

65.71%, 31.97%, 2.48%

Isolated mass = 114 mg

Figure S32 (2.0 eq. *n*-BuLi 2.5 M, 20 min, Gly:KF 6:1)

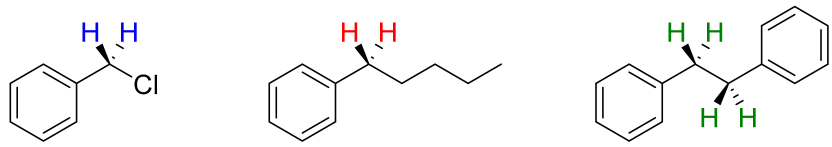

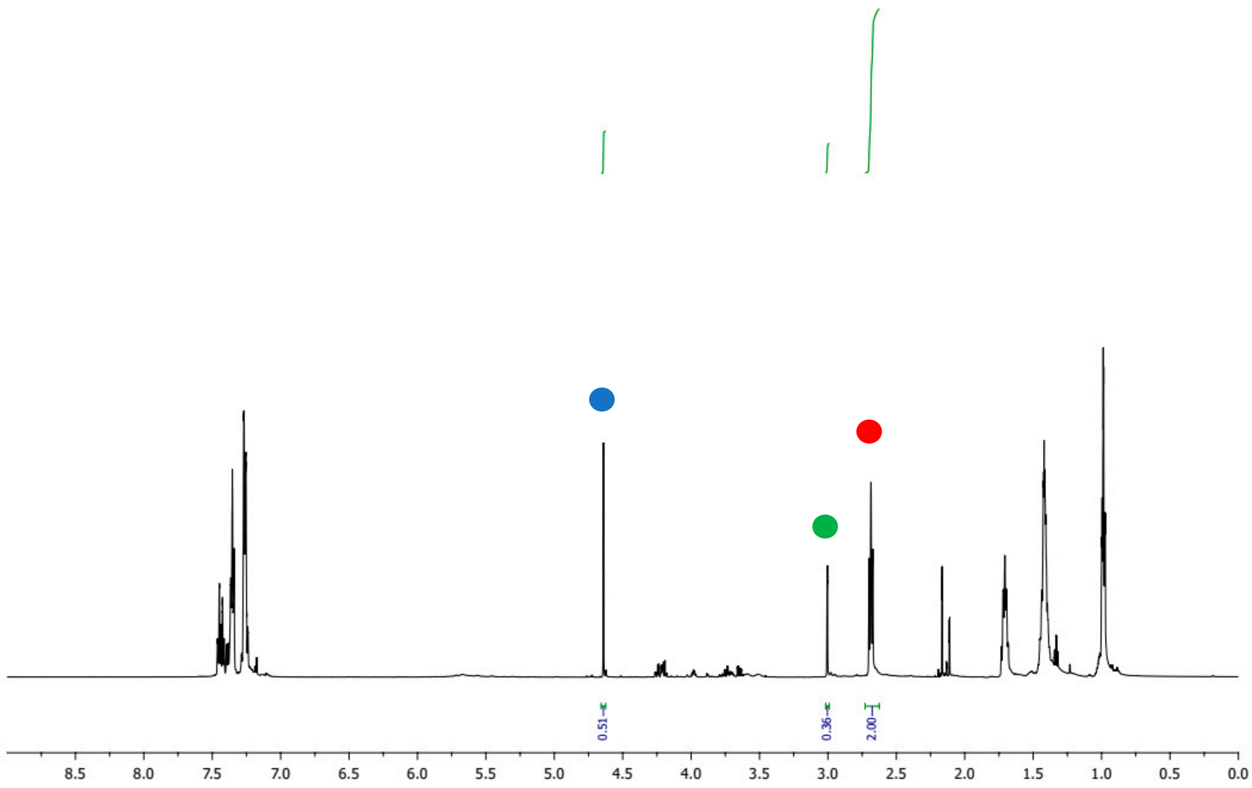

0.51 : 0.18 : 2.00

18.96%, 74.35%, 6.69%

Isolated mass = 127 mg

Figure S33 (2.0 eq. *n*-BuLi 2.5 M, 20 min, Gly:ChCl 2:1)

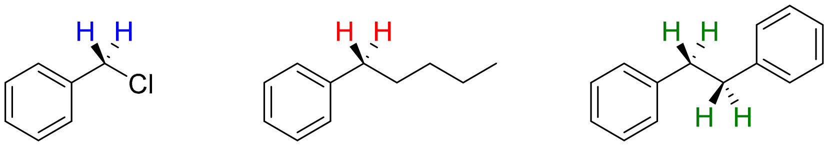

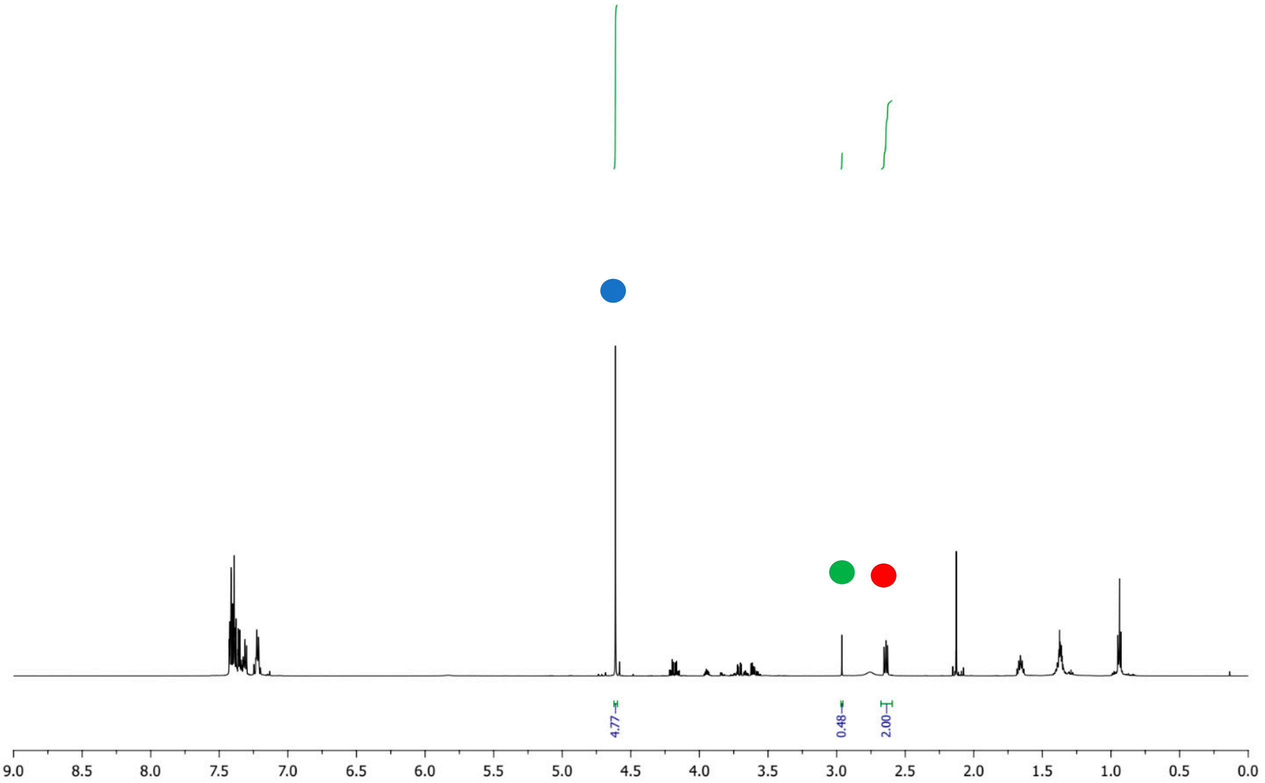

4.77 : **0.24** : **2.00**

68.05%, **28.53%**, **3.42%**

Isolated mass = 117 mg

Figure S34 (2.0 eq. *n*-BuLi 2.5 M, 20 min, Gly:ChCl 3:1)

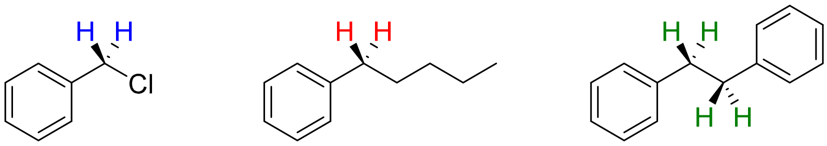

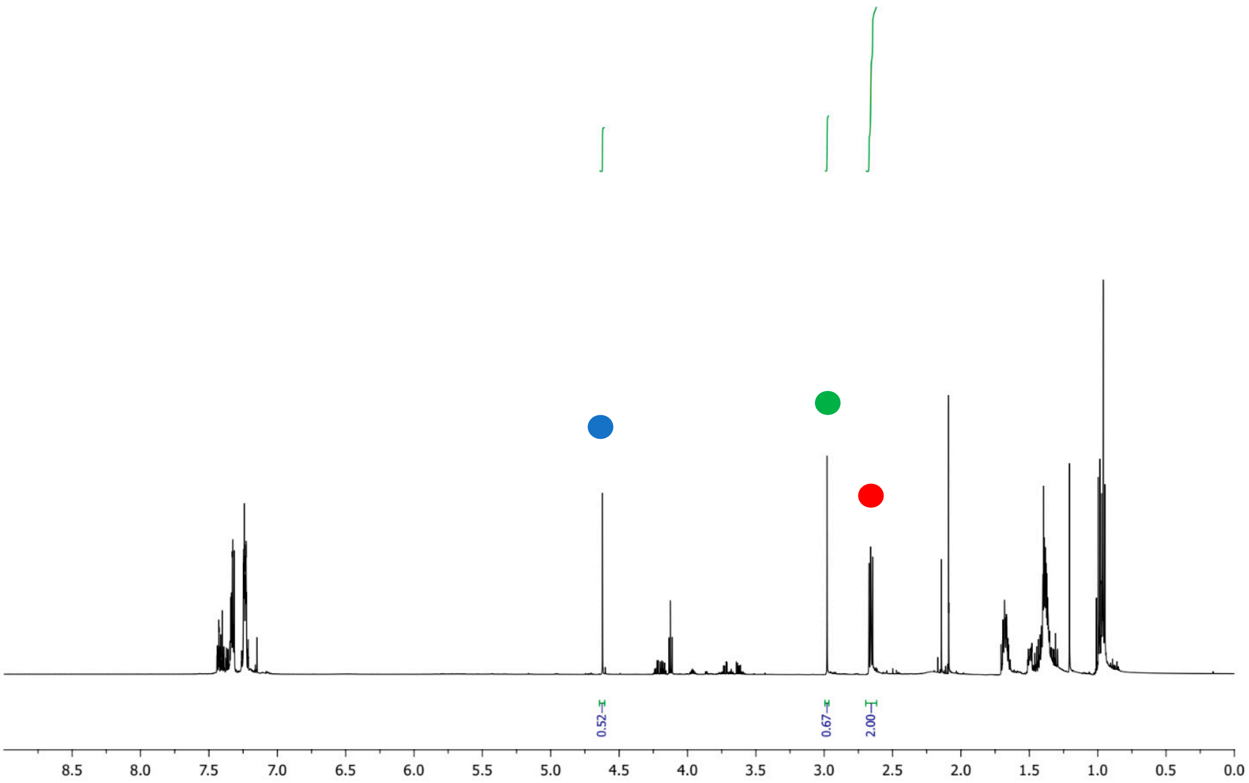

0.52 : 0.335 : 2.00

18.22%, 70.05%, 11.73%

Isolated mass = 151 mg

Figure S35 (2.0 eq. *n*-BuLi 2.5 M, 20 min, H_2O:ChCl 2:1)

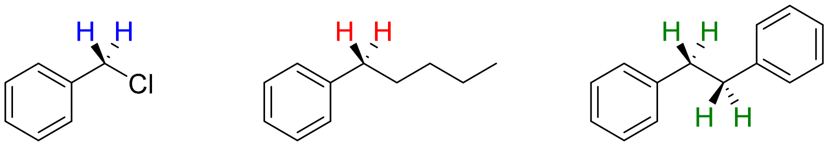

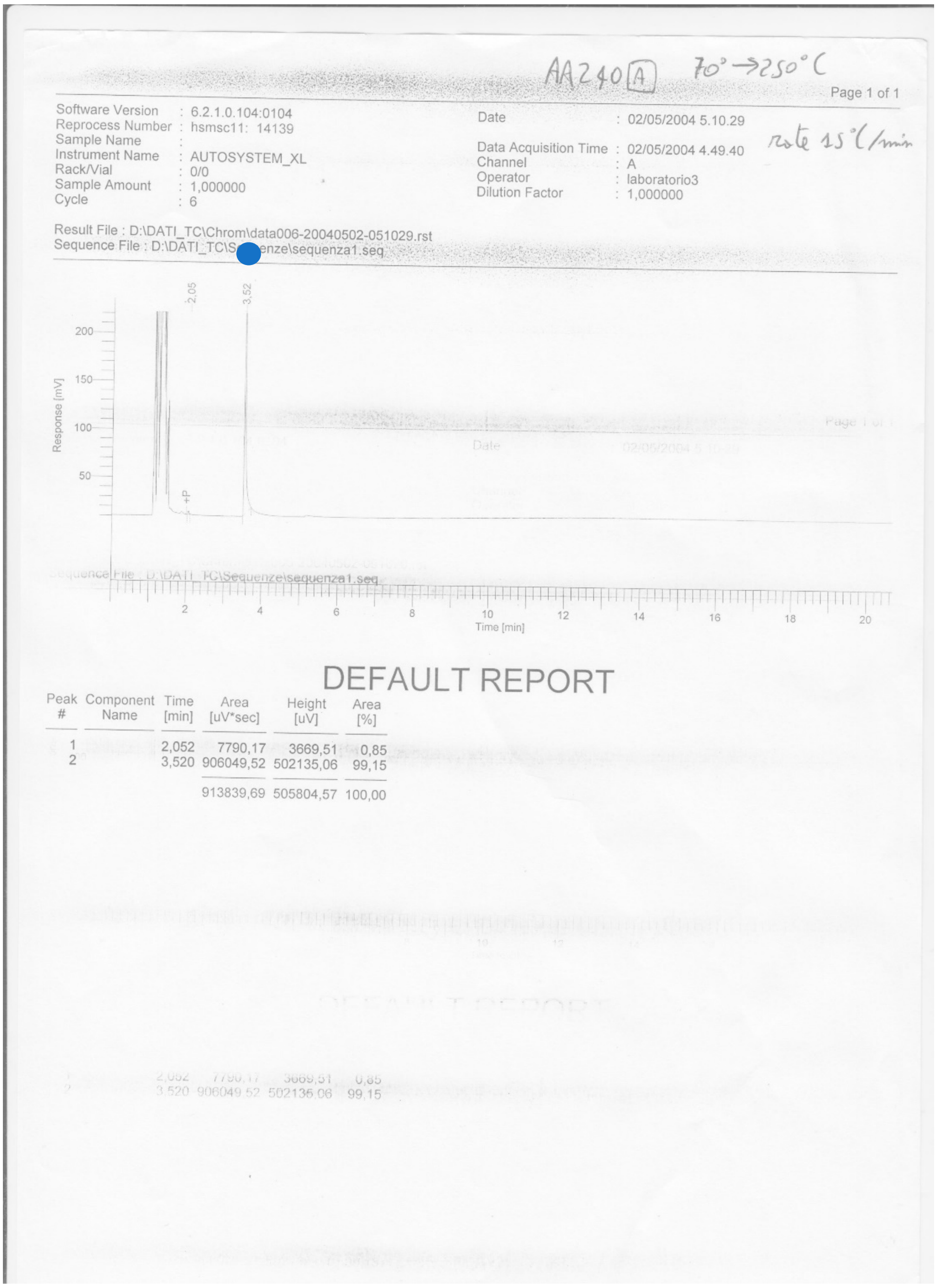

AA240 [A] 70° → 250°C

rate 15°C/min

Page 1 of 1

Software Version : 6.2.1.0.104:0104
Reprocess Number : hsmsc11: 14139
Sample Name :
Instrument Name : AUTOSYSTEM_XL
Rack/Vial : 0/0
Sample Amount : 1,000000
Cycle : 6

Date : 02/05/2004 5.10.29
Data Acquisition Time : 02/05/2004 4.49.40
Channel : A
Operator : laboratorio3
Dilution Factor : 1,000000

Result File : D:\DATI_TC\Chrom\data006-20040502-051029.rst
Sequence File : D:\DATI_TC\S[illegible]enze\sequenza1.seq

DEFAULT REPORT

Peak #	Component Name	Time [min]	Area [uV*sec]	Height [uV]	Area [%]
1		2,052	7790,17	3669,51	0,85
2		3,520	906049,52	502135,06	99,15
			913839,69	505804,57	100,00

100%

Figure S36 (2.0 eq. *n*-BuLi 2.5 M, 20 min, EG:ChCl 2:1)

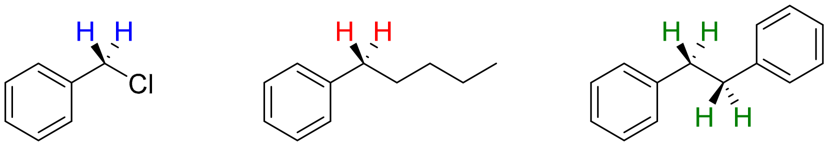

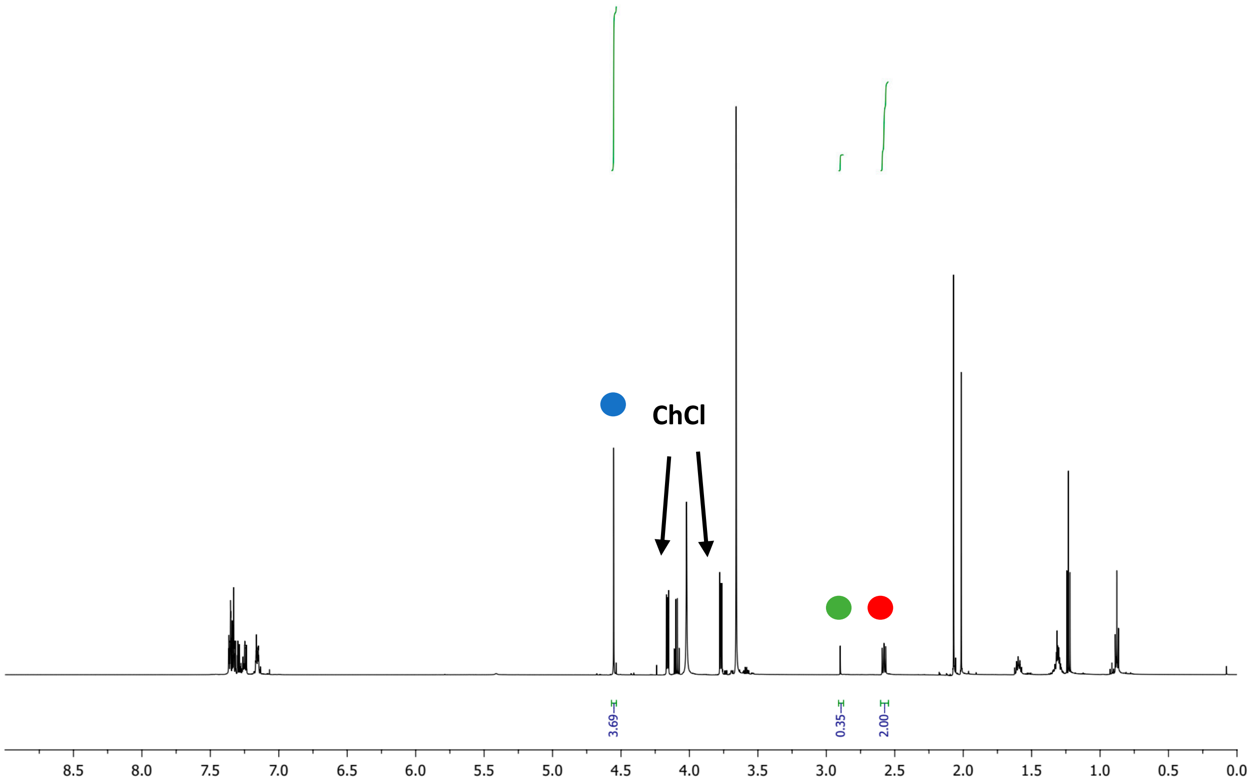

3.69 : 0.175 : 2.00

62.91%, 34.10%, 2.98%

Isolated mass = 187 mg

4. Bibliographic references

[1] [a] Abbott, A. P.; Capper, G.; Davies, D. L.; Rasheed, R. K.; Tambyrajah, V. *Chem. Commun.* **2003**, 70; [b] C. Vidal, C.; J. García-Álvarez, J.; A. Hernán-Gómez, A.; Kennedy, A. R.; Hevia, E. Introducing *Angew. Chem. Int. Ed.* **2014**, *53*, 5969-5973.

[2] Itami, K.; Saito, Y.; Segawa, Y.; Yamanoue, K. Selective Transformation of Strychnine and 1,2-Disubstituted Benzenes by C–H Borylation. *Chem* **2020**, *6*, 985-993.

[3] Maekawa, T.; Sekizawa, H.; Itami, K. Controlled Alcohol-Carbonyl Interconversion by Nickel Catalysis. *Angew. Chem. Int. Ed.* **2011**, *50*, 7022-7026.

[4] Condon, F. E; West, D. L. Cyclization-rearrangement of alkylstyrenes. 1. A. 1-Phenyl-1-pentene and homologs. B. A short synthesis of calamenene. *J. Org. Chem.* **1980**, *45*, 2006-2009.

[5] Ardiansah, B.; Tanimoto, H.; Tomohiro, T.; Morimoto, T.; Kakiuchi, K. Sulfonium ion-promoted traceless Schmidt reaction of alkyl azides. *Chem. Commun.* **2021**, *57*, 8738-8741.

[6] Matsui, M.; Tsuge, M.; Shibata, K.; Muramatsu, H. Photochromism of 1,1-Diaryl-1-alkanols. *Bull. Chem. Soc. Jpn.* **1994**, *67*, 1753-1755.

[7] Napolitano, E.; Giovani, E.; Ceccarelli, N.; Pelosi, P. Tertiary Alcohols with Earthy Odor. *J. Agric. Food Chem.* **1996**, *44*, 2806-2809.

[8] Gao, F.; Deng, X.-J.; Tang, Y.; Tang, J.-P.; Yang, J.; Zhang, Y.-M. A simple and efficient copper oxide-catalyzed Barbier-Grignard reaction of unactivated aryl or alkyl bromides with ester. *Tetrahedron Lett.* **2014**, *55*, 880-883.

[9] Ghinato, S.; Territo, D.; Maranzana, A.; Capriati, V.; Blangetti, M.; Prandi, C. A Fast and General Route to Ketones from Amides and Organolithium Compounds under Aerobic Conditions: Synthetic and Mechanistic Aspects. *Chem. Eur. J.* **2021**, *27*, 2868-2874.

[10] Le Bailly, B. A. F.; Greenhalgh, M. D.; Thomas, S. P. Iron-catalysed, hydride-mediated reductive cross-coupling of vinyl halides and Grignard reagents. *Chem. Commun.* **2012**, *48*, p. 1580-1582.

[11] Maeda, H.; Kikui, T.; Nakatsuji, Y.; Okahara, M. Synthesis of aminomethyl crown ethers. *J. Org. Chem.* **1982**, *47*, 5167-5171.
